# Genomic characterization of respiratory syncytial virus genotypes circulating in the paediatric population of Sydney, NSW, Australia

**DOI:** 10.1099/mgen.0.001095

**Published:** 2023-09-01

**Authors:** Krisna N. A. Pangesti, Hifzur R. Ansari, Ali Bayoumi, Alison M. Kesson, Grant A. Hill-Cawthorne, Moataz Abd El Ghany

**Affiliations:** ^1^​ School of Public Health, Faculty of Medicine and Health, The University of Sydney, Sydney, Australia; ^2^​ King Abdullah International Medical Research Center, King Saud Bin Abdulaziz University for Health Sciences, Jeddah, Saudi Arabia; ^3^​ The Westmead Institute for Medical Research, The University of Sydney, Sydney, Australia; ^4^​ Department of Infectious Diseases and Microbiology, The Children’s Hospital at Westmead, Sydney, Australia; ^5^​ Sydney Infectious Diseases Institute, The University of Sydney, Sydney, Australia; ^6^​ Discipline of Child and Adolescent Health, The University of Sydney, Sydney, Australia; ^7^​ The Westmead Clinical School, Faculty of Medicine and Health, The University of Sydney, Sydney, Australia

**Keywords:** Respiratory syncytial virus, molecular, epidemiology, genotype, whole-genome sequencing and NSW

## Abstract

Respiratory syncytial virus (RSV), or human orthopneumovirus, is a major cause of acute lower respiratory infection (ALRI), particularly in young children, causing significant morbidity and mortality. We used pathogen genomics to characterize the population structure and genetic signatures of RSV isolates circulating in children in New South Wales between 2016 and 2018 and to understand the evolutionary dynamics of these strains in the context of publicly available RSV genomes from the region and globally. Whole-genome phylogenetic analysis demonstrated the co-circulation of a few major RSV clades in the paediatric population from Sydney. The whole-genome-based genotypes A23 (RSV-A ON1-like genotype) and B6 (RSV-B BA9-like genotype) were the predominant RSV-A and RSV-B genotypes circulating during the study period, respectively. These genotypes were characterized with high levels of diversity of predicted N- and O-linked glycosylation patterns in both the G and F glycoproteins. Interestingly, a novel 72-nucleotide triplication in the sequence that corresponds to the C-terminal region of the *G* gene was identified in four of the A23 genotype sequenced in this study. Consistently, the population dynamics analysis demonstrated a continuous increase in the effective population size of A23 and B6 genotypes globally. Further investigations including functional mapping of mutations and identifying the impact of sequence changes on virus fitness are highly required. This study highlights the potential impact of an integrated approach that uses WG-based phylogeny and studying selective pressure events in understanding the emergence and dissemination of RSV genotypes.

## Data Summary

The genome sequences generated in this study have been submitted to NCBI ENA under study accession numbers PRJEB40107 (RSV-A) and PRJEB40108 (RSV-B). The genome assembly with annotation have been also deposited in the NCBI GenBank under accession numbers (OQ941653–OQ941776). The authors confirm all supporting data, code and protocols have been provided within the article or through supplementary data files.

**Impact Statement**

This study highlights the advantage of using WG-based genotyping, instead of *G* sequence, in distinguishing between RSV strains. The study also highlights the need to accompany this with the study of selective pressure events in G and F proteins and the potential implications on fitness advantage of emerging RSV strains. This includes glycosylation pattern and sequence duplication in G protein sequences, with a novel duplication pattern identified in some of the strains sequenced in this study. The G and F proteins are both essential for infection; the G protein is required for attachment while the F protein is required for membrane fusion. The F protein, which is more genetically conserved, is the main target for neutralizing antibodies induced by natural infection while the G protein has very wide genetic variation. The glycosylation of these two proteins, to which the immune response is directed, may mask neutralizing epitopes and be important for understanding immune escape. We hypothesize that genetic variability along with information of predicted glycosylation changes of the G and F proteins will be important in understanding RSV evolution and the design of vaccines and anti-viral agents that are needed to prevent infection or treat established infection.

## Introduction

Acute lower respiratory infection (ALRI) is one of the leading causes of death globally among children under 5 years of age [1, 2]. Human respiratory syncytial virus (RSV), also known as human orthopneumovirus [3], is the most frequent viral cause of ALRI in children worldwide [4] and is increasingly recognized as an important pathogen in elderly people and immunocompromised patients [5, 6]. Globally, it has been estimated that 33 million RSV-ALRI cases and approximately 118 000 deaths occur annually in children younger than 5 years [4] with approximately 67 % (22 million) of cases and 87 % (103,000) of deaths reported from low- and middle-income countries [4]. Estimates suggest that one in every 50 deaths in children aged 0–60 months and one in every 28 deaths in children aged 28 days to 6 months may be attributed to RSV infections [7]. The RSV-associated disease burden among children under 5 years in 72 Global Alliance for Vaccines and Immunisation (GAVI) countries has been estimated at 20.8 million cases, 1.8 million hospital admissions and 40 000 deaths [8]. These RSV infections resulted in 1.2 million discounted disability-adjusted life-years (DALYs), and US$611 million in discounted direct costs [8].

A recent study has estimated that RSV infection accounted for 1.5 million episodes of ALRI in older adults in high-income countries, with approximately 15 % (214,000) of these cases admitted to hospital [9]. The global number of hospital admissions and mortality associated with RSV-ALRI in older adults was estimated at 336 000 hospitalizations and 14 000 deaths, respectively [9].

There is currently no vaccine available against RSV, although several novel candidate vaccines are being tested in clinical trials [10, 11]. Passive immunization with monoclonal antibody against the RSV fusion protein (palivizumab) is the only approved prophylactic therapy for preterm infants and children at high risk from RSV infections [12]. No effective treatments for acute infection are currently available for RSV [13]. Recently, the World Health Organization (WHO) has launched the second phase of a global RSV surveillance programme to generate a robust understanding of the seasonality, risk groups, disease burden and viral genotypes of RSV, and provide evidence to support public health responses and to inform a future vaccination policy [14, 15].

RSV is an enveloped virus with an approximate 15 kb non-segmented, linear, single-stranded negative-sense RNA genome [16, 17]. The RSV genome encodes for 11 proteins. The proteins required for viral replication are the nucleocapsid protein [N], the phosphoprotein [P] and the large polymerase protein [L] and for transcription there are the regulatory proteins [M2-1 and M2-2] [18, 19]. Viral assembly uses the structural matrix protein [M], the attachment glycoprotein [G], and the fusion glycoprotein [F] [20, 21]. For attachment and entry into host cells, the G and F proteins, and a small hydrophobic protein [SH] are required [22–24]. Evasion of the host immune responses is mediated by the non-structural proteins-1 and −2 [NS1 and NS2], which impair interferon activity [25, 26], and the truncated secretory form of the G protein acts as decoy for the neutralizing antibodies of Fc receptor-bearing lymphocytes [27].

RSV is classified into two subgroups, A (RSV-A) and B (RSV-B), according to antigenic reactivity and these are further classified into genotypes based on genetic variation within the highly variable G gene [28].

Molecular characterization and identification of the evolutionary patterns of the G protein have provided important information on the transmission dynamics of RSV, including the co-circulation of different genotypes of both subgroups during epidemics [28, 29] and the displacement of dominant genotypes over successive epidemic seasons [30, 31]. A number of studies have reported the capability of particular genotypes of RSV to predominate within a community [32–34], causing recurrent [35, 36] and severe infections [37–39]. These have mainly been attributed to mutations in the G protein [40]. However, a recent study has suggested a potential role for mutations in the *F* and *L* genes in establishing repeated RSV-B infections in young children [41]. Several genotype classification schemes based either on part of (HVR2 [42] or Ectodomain [43]) or the entire G gene sequence [44, 45], have been used to define RSV genotypes. However, these genetic determinants provide insufficient phylogenetic signal and therefore are not ideal for constructing reliable phylogenies. Recently a proposed WG-based scheme, that used bootstrap support (BS) values and patristic distances, has distinguished 23 genotypes within RSV-A (designated A1–A23) and six genotypes within RSV-B (designated B1–B6) [46].

The incidence of childhood pneumonia in the Western Pacific region (WPR) of the WHO was estimated at 0.11 episodes per child-year with the contribution of viral pathogens, including RSV, still unclear [47]. A recent systematic review and meta-analysis found that RSV accounted for approximately 17 % of the total ALRI cases in WPR [48]. In Australia, the RSV epidemiology and laboratory data are state-based and mostly collected as part of research studies or laboratory network reports. The estimated RSV incidence in Australia was found to be higher in young children less than 24 months of age with a range from 110 to 226 per 1000 children under 5 years to 435–869 per 1000 infants [49, 50]. The RSV-associated hospitalization rate in children less than 5 years was estimated to be 418 per 100 000 population [49]. Recent phylogenetic analysis studies of RSV collected from New South Wales (NSW) and other states in Australia have demonstrated the complex dynamics of RSV infections and the rapid evolution of a diverse population of RSV-A and RSV-B subtypes at mean rates of approximately 6–8×10^−4^ nucleotide substitutions per site per year [51, 52]. A recent study has demonstrated a modification in the RSV epidemic’s seasonality in Australia after easing COVID-19 restrictions in late 2020, with unprecedented widespread outbreaks occurring in NSW and Western Australia in spring and summer 2021 [53].

In our study, we used whole-genome sequencing and bioinformatics to characterize RSV strains circulating in the paediatric population from Sydney, Australia during the recent successive seasons of 2016, 2017 and 2018. We comprehensively characterized the population structure and genetic signatures of the circulating RSV strains. We also aimed to better understand the epidemiological features and evolutionary dynamics of RSV from Sydney in the context of publicly available genomes both from the region and globally.

## Methods

### Study population

This study was performed on archived respiratory clinical specimens (nasopharyngeal swabs [NPS]) collected from children who tested positive for RSV and were admitted to The Children’s Hospital at Westmead (the biggest children’s hospital in NSW, located in Sydney (with 50 % of the state population resident) with acute respiratory infection between January 2016 and December 2018. RSV-positive samples were identified using multiplex reverse transcription PCR (RT-PCR) Seegene RV15 ACE Detection (Seegene, Cat No. RV6F01Y) during 2016–2017 and multiplex RT-PCR Seegene Allplex Respiratory Panel 1 (Seegene, Cat No. RP9801X) during 2018. The samples were stored at −80 °C until RNA was extracted. The samples with low qRT-PCR cycle threshold values (≤20) or those collected from children (less than 2 years old) with increased disease duration (> 5 days) and inpatients who had been admitted to the ICU were selected for genomic characterization.

### RNA extraction and RT-PCR

Total RNA was extracted from 140 µl of NPS specimen using QIAmp viral RNA mini kit (Qiagen, Hilden, Germany) according to the manufacturer’s instructions. The extracted RNA was concentrated sixfold (60 to 10 µl) using a centrifugal vacuum concentrator (Eppendorf 5301). RNA was quantified and stored at −80 °C until use.

RT-PCR was performed using previously published [54] genotype-specific primers that amplify the genome into six overlapping fragments of approximately 2.5 kb each (Table S1, available in the online version of this article). Complete RSV-A and RSV-B genomes available in GenBank (as of late February 2020) were searched using the Basic Local Alignment Search Tool (blast) and manually checked to identify polymorphisms in primer target sequences. The primers, with no or minimal polymorphisms in the primer target sequences in the publicly available genomes, were selected for use in RT-PCR. The first strand cDNA was synthesized using a GoScript RT Kit (Promega, Madison, Wisconsin, USA). The resultant cDNA was then split across six parallel PCR reactions to amplify RSV amplicons using GoTaq Long PCR Master Mix (Promega, Madison, WI, USA) (Table S1). The RSV amplicons were verified on a 1 % agarose gel, the concentrations were then quantified using Nanodrop and all six amplicons per genome were pooled in equal concentration.

### Library preparation and generating sequencing data

Preparation of sequencing libraries and the generation of sequencing data were performed at the Ramaciotti Centre for Genomics, University of New South Wales, Sydney, Australia. Briefly, the sequencing libraries were prepared using Nextera XT DNA library prep kits (Illumina, San Diego, CA, USA) according to the manufacturer’s protocol. The concentrations of individual libraries were measured using fluorometric methods and/or bead-based normalization. Equal molar concentrations of the individual libraries were pooled and paired-end sequencing was performed on an Illumina NovaSeq 6000 SP Flow cell 2×150 bp according to the manufacturer’s protocol.

### RSV genome assembly and annotation

An in-house integrated pipeline for assembly, annotation and visualization of the viral genomes was developed and used in this study. Briefly, the raw paired-end reads were trimmed using Trimmomatic v0.38 with default parameters [55]. The trimmed reads were mapped to a human genome and/or custom full-length RSV nucleotide database using Bowtie2 v2.3.5 [56] to identify host contaminants or RSV-specific reads in every sample. The RSV-specific reads (human unmapped reads) were extracted using SAMtools v1.8 [57] and assembled *de novo* using both Trinity v2.8.4 [58] and SPAdes v3.11.1 [59] with default parameters. The contigs generated from each assembler were processed separately. Contigs of slightly longer sizes and better statistics (e.g. N50 and L50 values) were generated using Trinity, therefore Trinity contigs/scaffolds were used for further analysis. The quality of the resulting multi-contigs was evaluated using quast v5.0.2 [60]. The trimmed reads were mapped back to the draft assembly using Bowtie2 v2.3.5 to identify the coverage of the resulting contigs. blast [61] was used to identify taxonomic classification. The resulting contigs were searched against custom full-length RSV nucleotide databases to identify the closest reference sequence. The contigs that had RSV sequence similarities of at least 90 % and an average coverage of greater than 2000-fold were selected and ordered manually using the closest RSV genome as a reference. The ordered contigs (draft assembly) were plotted and checked using MUMmer v4.0.0.2 [62] and BEDtools v2.29.0 [63]. Curated assemblies were validated and annotated using Viral Genome ORF Reader (VIGOR) v4.0 [64]. The annotated genomes were curated and visualized using Artemis [65, 66].

### Phylogenetic analysis

The Multiple Alignments using Fast Fourier Transform (MAFFT) programme was used to generate whole-genome and gene-specific alignments [67, 68]. Briefly, RSV-A and RSV-B sequences were aligned separately using the l-INS-I algorithm and all the alignments were checked and verified manually. Maximum-likelihood phylogenies were generated in RAxML-NG web tool [69] using a GTR +I+G nucleotide substitution model (time reversible model with a nucleotide site-specific gamma rate heterogeneity with four rate categories and invariant sites) and 1000 bootstrap replications. The dated trees and skyline plots were inferred using TreeTime tool [70]. The corresponding reference genomes, VR-26 (RSV-A) and VR-955 (RSV-B), and almost full RSV genomes (> 12 kb) available in GenBank as of late April 2021 were included in our analysis.

### Predictions of glycosylation sites

The glycosylation patterns of the surface proteins G and F were identified using NetNGlyc [71] and NetOGlyc [72] web tools. The glycosylation sequons and amino acid coordinates for the N-linked and O-linked glycans were extracted using custom PERL scripts and visualised in R using ggplot2 package [73].

## Results

### Demographic and clinical characteristics of RSV in 2016–2018 seasons

A total of 2455 NPS samples collected from children admitted to the Children’s Hospital at Westmead with acute respiratory infection between January 2016 and December 2018 tested positive for RSV. In total, 579 samples were available for further characterization in this study, of which 268(46.2 %) and 309(53.3 %) were identified as RSV-A and RSV-B, respectively. The distribution of the RSV-A and RSV-B positive cases during this period followed distinct seasonality patterns (Fig. S1). The peak of RSV cases occurred during April–May in 2016 and 2017 and in August for 2018. RSV-A and RSV-B subgenotypes were co-circulating in every year, with RSV-B being the predominant subgroup circulating in the 2017 season.

The demographic characteristics of the RSV cases in this study are shown in Table 1. RSV cases were mostly detected in males (55 %) aged less than 2 years old (86 %). The distributions of RSV-A and RSV-B in both categories (male and age less than 2 years old) were similar. The mean age of RSV-A was 1.02 years (range 0.3 months to 14.97 years) and RSV-B was 1.23 years (range 0.3 months to 15.91 years). We also found that most of the positive RSV tests (72.1 %) were taken within 1–3 days of hospital admission. The average length of stay for RSV-A positive cases was 5.5 days (range 0–176 days) and for RSV-B was 3.98 days (range 0–111 days). The samples submitted for whole-genome sequencing (WGS) were belonged mostly to children less than 2 years old.

**Table 1.** Demographic and clinical characteristics of RSV cases enrolled in this study

	2016						2017						2018				Total	
	RSV-A		RSV-B		Mixed		RSV-A		RSV-B		Mixed		RSV-A		RSV-B		Mixed	
	**n**	**%**	**n**	**%**	**n**	**%**	**n**	**%**	**n**	**%**	**n**	**%**	**n**	**%**	**n**	**%**	**n**	**%**
**Sex**																		
Male	34	22.2	34	20.4	1	50	30	19.6	63	37.7	1	50	89	58.2	70	41.9	322	55.6
Female	21	18.3	23	16.2	0	0	32	27.8	49	34.5	0	0	62	53.9	70	49.3	257	44.4
**Age group**																		
0–2	51	21.9	45	17.1	1	50	51	21.9	103	39.2	1	50	131	56.2	115	43.7	498	86
2–5	3	10.7	11	30.6	0	0	10	35.7	9	25	0	0	15	53.6	16	44.4	64	11.1
more than 5	1	14.3	1	10	0	0	1	14.3	0	0	0	0	5	71.4	9	90	17	2.9
**Length of stay (days)**																		
1–3	39	21.1	45	19.4	0	0	39	21.1	79	34.1	1	100	107	57.8	108	46.6	418	72.2
4–7	11	20.8	8	15.7	0	0	18	34	20	39.2	0	0	24	45.3	23	45.1	104	18
more than 7	5	16.7	4	15.4	1	100	5	16.7	13	50	0	0	20	66.7	9	34.6	57	9.8

### Characterizing RSV genomes from Sydney, NSW

Overall, 106 samples (55 belonging to RSV-A and 51 to RSV-B) from the 2016–2018 seasons were selected for whole-genome sequencing based on viral load in clinical samples (qRT-PCR cycle threshold value <20) and disease severity (children attending the hospital with increased disease duration and/or ICU admission). Nearly complete RSV genomes were generated for all the samples sequenced in this study. The length of each genome ranged from 14 000 to 15 100 nucleotides, and the average depth of coverage ranged from 2000- to 7000-fold. The ON1 genotype, characterized by a 72-nucleotide duplication in the G gene, was the predominant strain identified in 94 %(48 out of 51) of RSV-A genomes sequenced in this study, with NA1 genotype detected in six genomes (one in 2017 and five in 2018). The recently emerged BA9 genotype was identified in all RSV-B genomes from the 2016–2018 seasons.

### Global analysis of RSV

Whole-genome-based maximum-likelihood phylogenies of the RSV-A and RSV-B genomes sequenced in this study along with the corresponding reference RSV genomes available in GenBank (as of late April 2021) are shown in Figs S2 and S3. The RSV strains clustered into major clades that included genotypes GA1-7 and NA1 (RSV-A) and GB1-5 and BA (RSV-B). The global RSV-A and RSV-B phylogenies were dominated by the recently evolved ON1 (introduced in 2011) and BA9 (introduced in 2002) strains, respectively.

### Genetic evolution of RSV strains from NSW

**Fig. 1.**
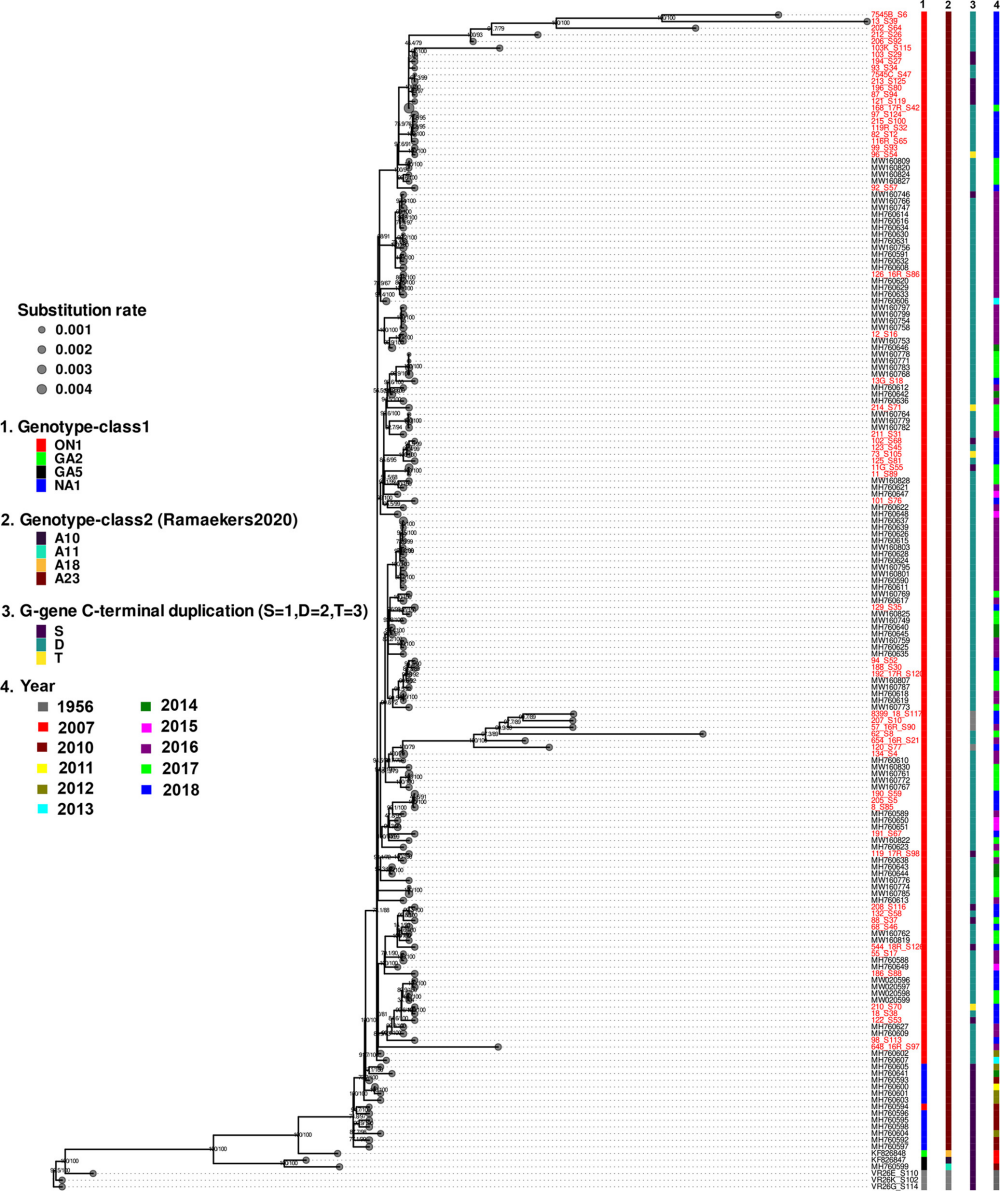
Phylogeny of respiratory syncytial virus subtype A strains from NSW. A mid-point rooted whole-genome-based maximum-likelihood phylogenomic tree of RSV-A strains sequenced in this study (taxa highlighted in red) and the corresponding publicly available RSV genomes from Australia [51, 52]. Colour-coded information on each strain is illustrated to the right that include genotype identified based on G protein gene sequence (column 1) and whole-genome sequencing (column 2) and sequence duplication pattern in the C-terminus of the G gene (column 3). The substitution rate is illustrated by grey circles.

**Fig. 2.**
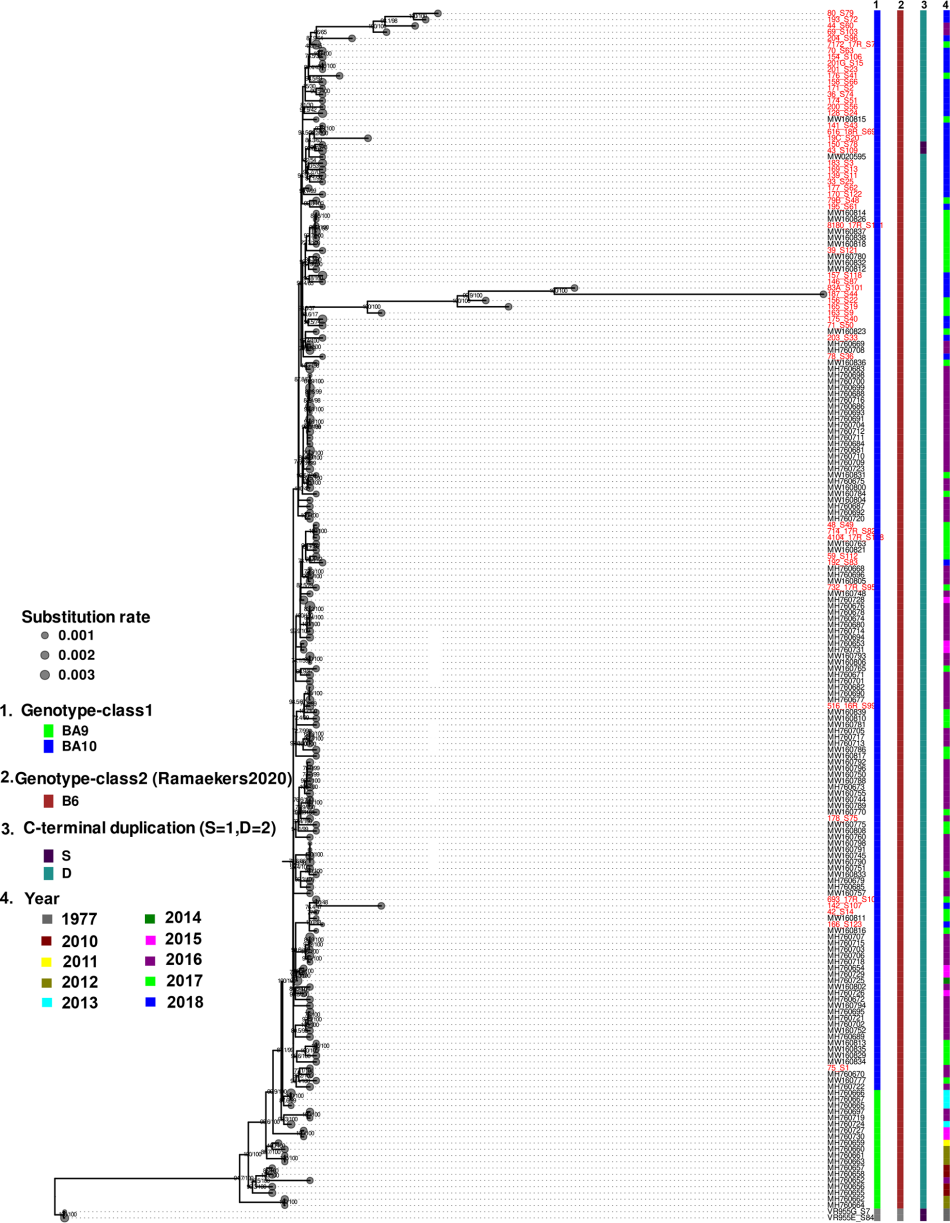
Phylogeny of respiratory syncytial virus subtype B strains from NSW. A mid-point rooted whole-genome-based maximum-likelihood phylogenomic tree of RSV-B strains sequenced in this study (taxa highlighted in red) and the corresponding RSV genomes from Australia [51, 52]. Colour-coded information on each strain is illustrated to the right that include genotype identified based on G protein gene sequence (column 1) and whole-genome sequencing (column 2) and sequence duplication pattern in the C-terminus of the G gene (column 3). The substitution rate is illustrated by grey circles.

Whole-genome-based maximum-likelihood phylogenies of RSV-A and RSV-B from NSW including the genomes sequenced in this study and other RSV genomes from Di Giallonardo *et al*. [51] and Robertson *et al*. [52] are shown in Figs 1 and 2. The majority of RSV-A and RSV-B genomes from NSW, particularly the most recently sequenced circulating strains in this study, clustered into multiple subclades of ON1 and BA9, respectively.

The RSV-A genomes clustered into two major clades. The RSV-A NA1 strains circulating in the period 2010–2013 fell into one clade, while RSVA ON1 (2012–2018) strains belonged to multiple subclades (branches) within the dominant clade (Fig. 1).

RSV-B genomes clustered into two major clades with multiple subclades (branches). The majority of RSV-B BA9 strains clustered into subclades according to the time of isolation. RSV-B BA9 circulating in the period 2012–2016 and two genomes of RSV-B BA10 genotype from the 2012 season fell within two closely related subclades within one of the major clades (Fig. 2).

### Population structure of WG-based RSV genotypes

We conducted a clustering analysis using the recently proposed criterion that defined RSV strains according to their position in the WG-based phylogeny (bootstrap support of >70 % and a maximum patristic distance of <0.018 substitutions per site between all clade members) [46]. The RSV-A genomes we studied could be distinguished into four WG-based genotypes (A10, A11, A18 and A23). Interestingly, the A23 genotype was the dominant type that comprised both the RSV-A ON1 and RSV-A NA1 lineages (Fig. 1). Four of the A23 genotype (RSV-A ON1) contained a novel 72-nucleotide triplication (23-amino acid triplication) in the sequence that corresponds to the C-terminal region of the G gene (Figs 1 and S4). The novel repeated sequence was confirmed using *in silico* analysis. The distribution and depth of sequence reads that mapped back to the duplication region in the G gene of the four assembled genomes are shown (Fig. S5).

Interestingly, all RSV-B genomes were assigned identical WG-based genotype (B6) based on the recently proposed criterion (bootstrap support of >70 % and a maximum patristic distance of <0.026 substitutions per site between all clade members) [46] that defines RSV genomes according to WG-based phylogeny (Fig. 2). All RSV-B genomes were of the globally dominating genotype characterized by a 60-nucleotide duplication located in the second variable region of the G gene (Figs 2 and S6). Interestingly, some recently circulating RSV-B genomes, including those that were sequenced in this study and other strains from NSW, share a highly conserved sequence motif (TQKLQSYA [dominant], TQRLQSYA [identified in one genome]) that flanks the amino acid 311 in the G protein (Fig. S6).

The clustering of the WG-based genotypes seems to fit with the topology of RSV-A and RSV-B phylogenetic trees. In contrast, RSV G gene-based genotypes clustered in multiple subclades within the same clade. Therefore, the inconsistencies identified between relatedness based on whole-genome sequencing versus G gene genotypes (as a measure of relatedness based on G protein gene diversity) is likely attributed to the high level of diversity in the G protein gene compared with the other regions of the genome. For the RSV genomes included in this analysis, the relatedness of the genotypes assigned by WGS and G gene are shown in Table S2.

### Glycosylation analysis reveals distinct glycosylation patterns in G and F proteins

**Fig. 3.**
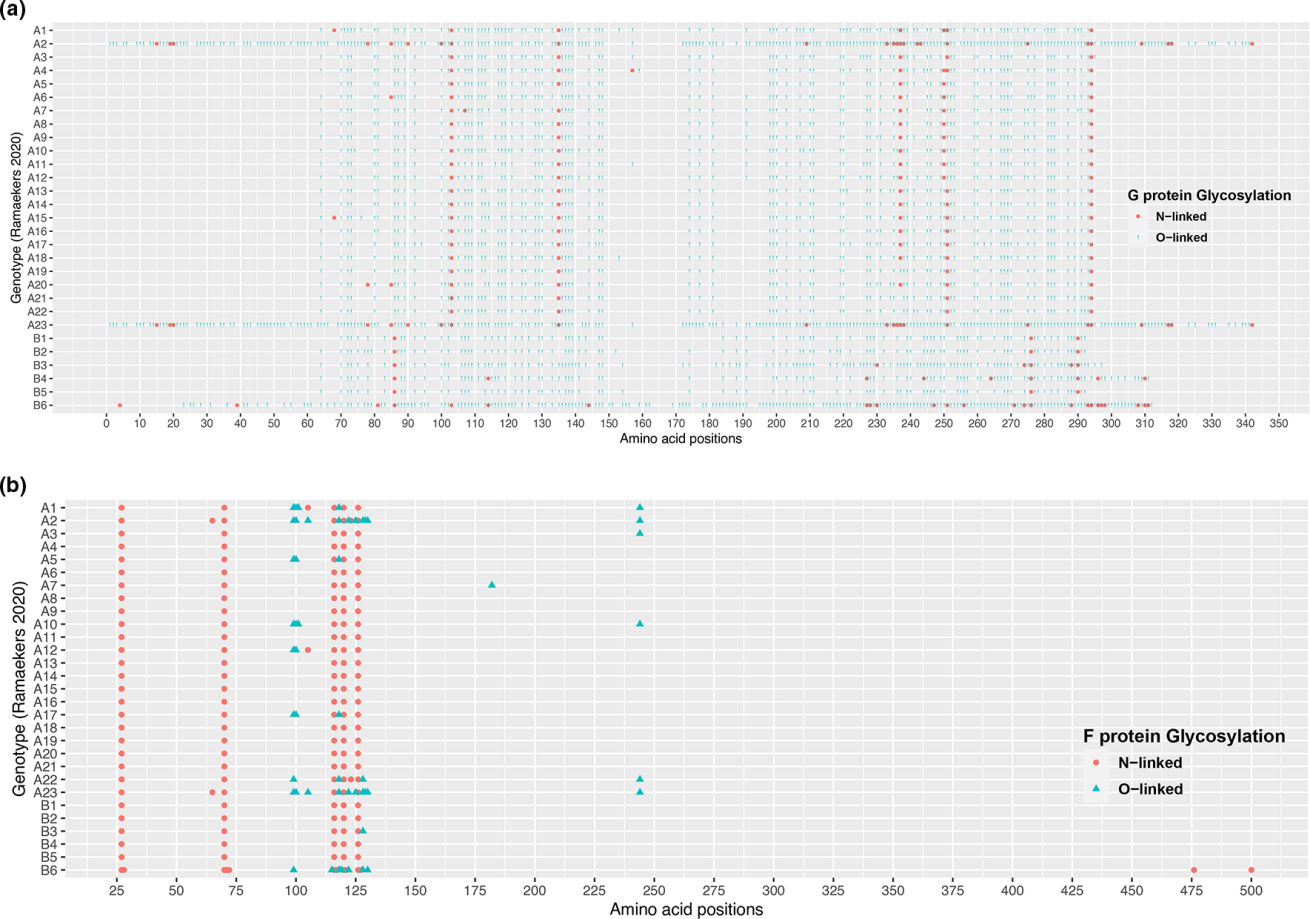
O- and N- linked glycosylation signatures in RSV subgroups. The genotype-specific consensus glycosylation patterns for O- and N-linked glycans of G protein (**a**) and F protein (**b**) are displayed in rows.

The glycosylation analyses demonstrate that almost all the RSV genomes analysed in this study shared a conserved pattern of predicted N- and O-linked glycosylation sequons in the F proteins and wide diversity of predicted N- and O-linked glycosylation sequons in the G gene. The distributions of O- and N-glycosylation patterns/signatures in RSV subgroups are shown in Fig. 3. Interestingly, the predominant genotypes in NSW (A23 and B6) were characterized with a high level of diversity related to the N- and O-linked glycosylation patterns in both the G and F glycoproteins (Fig. 3). Further investigations are required to characterize the structural and functional modifications of G and F glycoproteins associated with the predicted glycosylation signatures. These include *in vivo* studies to characterize potential roles of glycosylation signatures in viral replication, antigenicity and interactions with the host (e.g. persistence, transmissibility and immune evasion).

### Population dynamics analysis

**Fig. 4.**
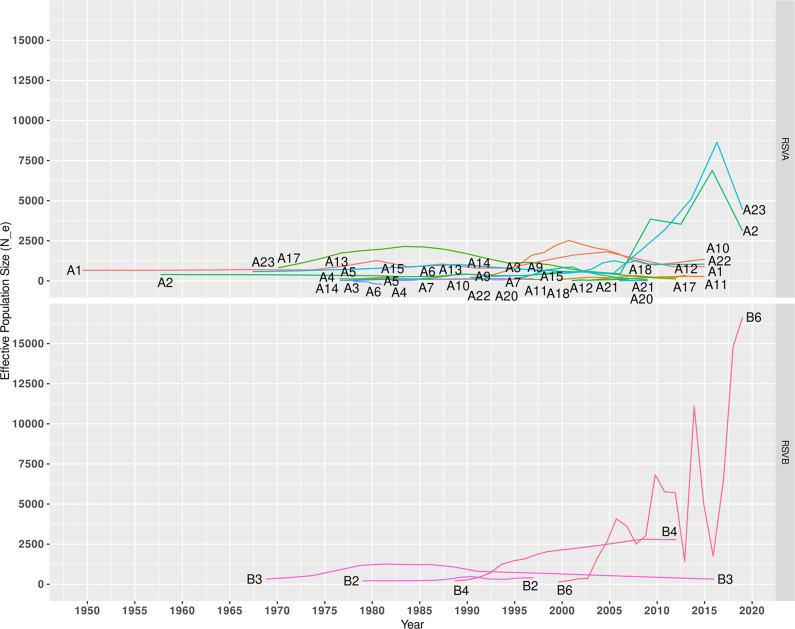
Population dynamics of whole-genome-based genotypes of RSV. Bayesian skyline plot method was used to represent the population dynamics of RSV. The effective population size and time (years) were represented on X- and Y-axis, respectively.

Population dynamics analysis revealed that the WG-based genotypes A2/A23 and B6 were the predominant RSV-A and RSV-B genotypes, respectively, circulating globally (Fig. 4). The Bayesian skyline plots demonstrated a continuous increase in the effective population size of A2 and A23 during 2005–2017, and B6 during 2005–2020 (Fig. 4). The continuous rise of the effective population size of these genotypes can be attributed to the fitness advantage gained due to the accumulation of selective pressure signatures/events in the most recently emerged RSV subtypes globally, including RSV-A ONA1 and RSV-B BA10. However, further investigations are required to confirm selective pressure patterns and identify the associated impact on the emergence and persistence of RSV subtypes/genotypes.

## Discussion

This recent study investigated the molecular characterization of RSV strains circulating in children attending The Children’s Hospital at Westmead in Sydney, which is the largest children’s hospital in New South Wales, Australia. RSV is not a notifiable disease in Australia, however, there is a network between laboratories that reports the number of RSV-positive cases regularly. There is little information available on the genetic diversity and the dynamic evolution of RSV within specific communities in Australia. Importantly, the molecular basis of the persistent RSV genotypes circulating within susceptible populations over consecutive epidemic seasons is still unclear.

Recently, several studies have highlighted the importance of conducting large-scale genomic analyses of RSV to provide comprehensive and accurate information on dynamics and evolution, which can assist in the control of disease transmission [17, 74]. High-throughput approaches, including WG-based metagenomic sequencing [75], target enrichment sequencing [76] and PCR amplicon sequencing [54], have been used to characterize RSV isolates/samples [77–79]. However, the sequencing of RSV viruses is challenging due to multiple factors, including the low abundance of viral load in clinical samples [80, 81], the high diversity of RNA genomes (due to high mutation rate) [82] and the requirement for reverse transcription (RT) and PCR amplification [83].

PCR amplicon sequencing, with amplicons either generated using specific primers [51–53] or through sequence-independent single primer amplification (SISPA) [77, 79, 84, 85], seems to have advantages over the other approaches, particularly in clinical samples with low viral loads [79]. However, a few studies have reported low success rates (60–67 %) in producing whole-genome-sequencing data from RSV samples [51, 74]. In this study, we employed an overlapping RT-PCR approach followed by whole-genome sequencing to generate nearly complete RSV genomes for all the studied samples. High quantities of RSV amplicons of correct fragment size were consistently obtained using genotypic-specific primers (six pairs of primers per subgroup were used to amplify six overlapping RSV fragments of 2.5 kb each), whose target sequences were conserved (i.e. no or minimal polymorphisms were detected in the primer target sequences in the publicly available genomes).

The analyses demonstrated that a few major RSV clades were co-circulating in the paediatric population from Sydney during the 2016–2018 seasons. It has been well-established that RSV-A and RSV-B can co-circulate together in the same epidemic season, with the shifting predominance of each occurring between years. In this study, RSV-A and RSV-B co-circulated during the 3 years of study, with RSV-B clearly predominant in 2017. The RSV-A ON1 and RSV-B BA9 clades were the predominant strains circulating during these epidemic seasons. RSV-A ON1 strains showed relative temporal clustering by year of detection.

A recent study has reported the circulation of a wide diversity of RSV lineages in a small geographic region within NSW during the period 2011–2016 [51]. These highlight the need to establish a robust surveillance for RSV to overcome the highly biassed sampling that hinders the accurate detection of the transmission dynamics of RSV.

The topologies of the global and Sydney RSV-A and RSV-B phylogenetic trees clearly demonstrate that the current system of genotyping based on the G protein gene sequence lacks discrimination between genotypes, with the genotypes overlapping with each other [30]. Consistently, several studies have demonstrated the potential impact of implementing molecular surveillance that use WG-based approaches to characterize genotypes and monitor the emergence and transmission of RSV strains [46, 86]. A recent study has suggested WGS as the most powerful tool to be used in assigning/revising RSV genotypes [87]. In another study, WG analysis has been shown to provide the best phylogenetic resolution compared with the sequences of the surface glycoprotein genes; SH, G and F (single or concatenated) [88].

The RSV F and G proteins are primary targets for protective immune responses [22, 89]. O- and N-glycosylation are essential to maintain the structural and functional integrity of these glycoproteins [89, 90]. These post-translational modifications affect both the antigenicity (to evade host immune responses) and virulence of RSV [91, 92]. Few studies have demonstrated a link between the occurrences of severe or recurrent infections and amino acid changes that alter the N- and O-glycosylation patterns of the F and G proteins [41, 93, 94]. Glycosylation prediction analysis demonstrated a high degree of variation in G protein sequences and relatively low variability in the F protein in both RSV-A and RSV-B strains sequenced in this study.

The F protein has a conserved glycosylation pattern across both RSV-A and RSV-B genotypes, with few O- and N-linked glycans being detected in strains recovered from young children. The RSV F protein shows 89 % homology among strains of different subtypes and is thereby the most conserved RSV envelope protein [95]. Previous studies have suggested that the F protein is required to retain functional fusion mechanisms, therefore high degree of conservation in the F1 domain is likely needed to maintain fitness [74].

The use of WGS to assign RSV genotypes and functional genomics to translate genotype to phenotype should provide important information not only for disease surveillance and epidemiology, but also vaccine and therapeutic development [86] .

This study provides insights on the population structure and the molecular diversity of RSV associated with paediatric population of Sydney, Australia. The study highlighted the importance of whole-genome data in understanding the transmission dynamics of HRSV. The study also highlighted the need to conduct further functional genomics investigations to identify the potential correlation between sequence changes and virus fitness and susceptibility to therapeutic and preventive interventions, including the functional mapping of mutations.

## Supplementary Data

Supplementary material 1
