# Supplementary material for: Genomic characterization of respiratory syncytial virus genotypes circulating in the paediatric population of Sydney, NSW, Australia
Source: Microb Genom. 2023 Sep 1;9(9):001095. doi: 10.1099/mgen.0.001095 (PMC10569731; doi:10.1099/mgen.0.001095)
Supplement: Supplementary material 1 [file mgen-9-1095-s001.pdf]

Supplementary Table 1. List of genotype-specific primers used in generating RSV amplicons

Primers	Sequence (5' –3')	RSV-A amplicons	Primers	Sequence (5' –3')	RSV-B amplicons
1F: rsva52	TGTGCATGTTATTACAAGTAGTGATATTTG	RSVAF1	1F:rsvb3	TGGGGCAAATAAGAATTTGATAAGTGC	RSVBF1
1R: rsva39	CTTCTCTTAAACCAACCATGGCATC		1R:rsvb50	AGTCTTGCCATAGCCTCTAACCT	
2F :rsva117	ATAAGAGATGCCATGGTTGGTTTAAGA	RSVAF2	2F:rsvb33	ATATTAGGAATGCTCCATACATTAGTAGTTG	RSVBF2
2R :rsva1644	CAACTCCATTGTTATTTGCCCC		2R:rsvb7423	CCATGGTTATTTGCCCCAGATTTAAT	
3F :rsva1820	GCAGCATATGCAGCAACAATC	RSVAF3	3F:rsvb7996	TATGTGGCAACAATCAACTCTGC	RSVBF3
3R :rsva312	AGGATATTTGTCAGGTAGTATCATTATTTTTGG		3R:rsvb3652	GCTTATGGTTATGCTTTTGTGGATATCTAAT	
4F :rsva704	ATGTGTTGCCATGAGCAAACTC	RSVAF4	4F:rsvb3762	AGAGGTCATTGCTTGAATGGTAGAA	RSVBF4
4R :rsva539	ATGCTTGATTGAATTTGCTGAGATCTG		4R:rsvb168	TGCATGTCTATATGTACATATTATTGTGACAAG	
5F :rsva374	AAGAGAACTCAGTGTAGGTAGAATGTTT	RSVAF5	5F:rsvb32	AAGAAGAGTACTAGAGTATTACTTGAGAGATAA	RSVBF5
5R :rsva385	ATCAGTTATATATCCCTCTCCCCAATCTT		5R:rsvb60	AACTTAAAACTGTGACAGCCTTTTATTCT	
6F :rsva1220	GATTGGGTGTATGCATCTATAGATAACAAG	RSVAF6	6F:rsvb165	TTCAAAATTGCATAAGTTTTGGTCTTAGC	RSVBF6
6R :rsva4066	GTTGTATAACAAACTACCTGTGATTTTAATCAG		6R:rsvb989	TATAGTACACTACCTGTTATTTTAATCAGCTTC	

Supplementary Table 2. Mapping WG-based and G gene based genotypes.

ID	Date	Country	Type	Genotype Class 1	Genotype Class 2 (Ramaekers2020)
AY353550.1	1977	USA	RSVB		
AY911262.1	1956	USA	RSVA		
FJ948820.1	1999	Netherlands	RSVA	GA2	A17
GU591758.1	2003	USA	RSVA		
GU591759.1	2003	USA	RSVA		
GU591760.1	2003	USA	RSVA		
GU591761.1	2003	USA	RSVA		
GU591762.1	2003	USA	RSVA		
GU591763.1	2004	USA	RSVA		
GU591764.1	2005	USA	RSVA		
GU591765.1	2003	USA	RSVA		
GU591766.1	2005	USA	RSVA		
GU591767.1	2003	USA	RSVA		
GU591768.1	2004	USA	RSVA		
GU591769.1	2004	USA	RSVA		
GU591770.1	2004	USA	RSVA		
GU591771.1	2004	USA	RSVA		
JF920046.1	2007	USA	RSVA	GA2	A22
JF920047.1	2008	USA	RSVA	GA2	A22
JF920048.1	2007	USA	RSVA	GA2	A22
JF920049.1	2007	USA	RSVA	GA2	A22
JF920050.1	2008	USA	RSVA	GA2	A22
JF920051.1	2007	USA	RSVA	GA2	A22
JF920052.1	2010	USA	RSVA	GA2	A23
JF920053.1	2010	USA	RSVA	GA2	A23
JF920054.1	2010	USA	RSVA	GA2	A23
JF920057.1	1998	USA	RSVA	GA2	A20
JF920058.1	2006	USA	RSVA	GA5	
JF920062.1	1998	USA	RSVA	GA7	A15
JF920065.1	1998	USA	RSVA	GA7	A15
JF920069.1	2006	USA	RSVA	GA1	
JQ582843.1	2002	USA	RSVB		B5
JQ582844.1	2002	USA	RSVB		B6
JQ901447.1	2001	Netherlands	RSVA	GA2	
JQ901448.1	2001	Netherlands	RSVA	GA5	A11
JQ901449.1	2001	Netherlands	RSVA	GA5	A11
JQ901450.1	2001	Netherlands	RSVA	GA5	A11
JQ901451.1	2001	Netherlands	RSVA	GA5	A10
JQ901452.1	2001	Netherlands	RSVA	GA2	A18
JQ901453.1	2002	Netherlands	RSVA	GA2	A18
JQ901454.1	2002	Netherlands	RSVA	GA5	A10
JQ901455.1	2002	Netherlands	RSVA	GA5	A10
JQ901456.1	2003	Netherlands	RSVA	GA5	
JQ901457.1	2003	Netherlands	RSVA	GA2	A20
JQ901458.1	2003	Netherlands	RSVA	GA2	A20

JX015479.1	2011	Netherlands	RSVA	GA2	A23
JX015480.1	2007	Netherlands	RSVA	GA2	A18
JX015481.1	2009	Netherlands	RSVA	GA2	
JX015482.1	2006	Belgium	RSVA	GA2	A23
JX015483.1	2008	Netherlands	RSVA	GA2	A23
JX015484.1	2007	Netherlands	RSVA	GA2	A23
JX015485.1	2005	Netherlands	RSVA	GA5	A10
JX015486.1	2005	Netherlands	RSVA	GA2	A20
JX015487.1	2006	Netherlands	RSVA	GA5	A12
JX015488.1	2006	Netherlands	RSVA	GA5	A10
JX015489.1	2007	Netherlands	RSVA	GA2	A23
JX015490.1	2007	Netherlands	RSVA	GA2	A23
JX015491.1	2007	Netherlands	RSVA	GA2	A23
JX015492.1	2007	Netherlands	RSVA	GA2	A23
JX015493.1	2008	Netherlands	RSVA	GA2	A23
JX015494.1	2008	Netherlands	RSVA	GA2	A23
JX015495.1	2008	Netherlands	RSVA	GA2	A23
JX015496.1	2008	Netherlands	RSVA	GA2	A23
JX015497.1	2008	Netherlands	RSVA	GA2	
JX015498.1	2008	Netherlands	RSVA	GA2	A23
JX015499.1	2008	Belgium	RSVA	GA2	A22
JX069798.1	2001	USA	RSVA	GA2	A17
JX069799.1	2001	USA	RSVA	GA2	A20
JX069800.1	1997	USA	RSVA		A15
JX069801.1	1998	USA	RSVA		A15
JX069802.1	1998	USA	RSVA		A10
JX069803.1	2000	USA	RSVA		A10
JX576729.1	2008	Belgium	RSVB	GB13	B6
JX576730.1	2008	Belgium	RSVB	GB13	B6
JX576731.1	2008	Belgium	RSVB	GB13	B6
JX576732.1	2006	Belgium	RSVB	GB13	B6
JX576733.1	2012	Netherlands	RSVB	GB13	B6
JX576734.1	2012	Netherlands	RSVB	GB13	B6
JX576735.1	2012	Netherlands	RSVB	GB13	B6
JX576736.1	2012	Netherlands	RSVB	GB13	B6
JX576737.1	2010	Netherlands	RSVB	GB13	B6
JX576738.1	2010	Netherlands	RSVB	GB13	B6
JX576739.1	2009	Netherlands	RSVB	GB13	B6
JX576740.1	2009	Netherlands	RSVB	GB13	B6
JX576741.1	2009	Netherlands	RSVB	GB13	B6
JX576742.1	2009	Netherlands	RSVB	GB13	B6
JX576743.1	2009	Netherlands	RSVB	GB13	B6
JX576744.1	2008	Netherlands	RSVB	GB13	B6
JX576745.1	2008	Netherlands	RSVB	GB13	B6
JX576746.1	2008	Netherlands	RSVB	GB13	B6
JX576747.1	2008	Netherlands	RSVB	GB13	B6
JX576748.1	2007	Netherlands	RSVB	GB13	B6

JX576749.1	2007	Netherlands	RSVB	GB13	B6
JX576750.1	2007	Netherlands	RSVB	GB13	B6
JX576751.1	2007	Netherlands	RSVB	GB13	B6
JX576752.1	2006	Netherlands	RSVB	GB13	B6
JX576753.1	2006	Netherlands	RSVB	GB13	B6
JX576754.1	2005	Netherlands	RSVB	GB13	B6
JX576755.1	2005	Netherlands	RSVB	GB13	B6
JX576756.1	2005	Netherlands	RSVB	GB13	B6
JX576757.1	2005	Netherlands	RSVB	GB13	B6
JX576758.1	2005	Netherlands	RSVB	GB13	B6
JX576759.1	2003	Netherlands	RSVB	GB13	B6
JX576760.1	2003	Netherlands	RSVB	GB12	B5
JX576761.1	2002	Netherlands	RSVB	GB13	B6
JX576762.1	2002	Netherlands	RSVB	GB13	B6
JX627336.1	2011	SouthKorea	RSVA		A23
KC731482.1	2011	India	RSVA		A23
KC731483.1	2011	India	RSVA		A23
KC978856.1	2011	China	RSVA		A23
KF530259.1	2006	SouthAfrica	RSVB		B6
KF530260.1	2005	Argentina	RSVA		A10
KF530261.1	2008	Germany	RSVA		A23
KF530268.1	2007	Mexico	RSVA		A12
KF530269.1	2009	USA	RSVA		A23
KF640637.1	2005	UnitedKingdom	RSVB	BA9	
KF826819.1	2009	Italy	RSVB		B6
KF826820.1	2009	Italy	RSVB		B6
KF826821.1	2007	USA	RSVA		A22
KF826822.1	2007	USA	RSVB		B6
KF826823.1	1998	USA	RSVA		A10
KF826824.1	1998	USA	RSVA		A10
KF826825.1	2004	Mexico	RSVB		B6
KF826826.1	2004	Mexico	RSVA		A12
KF826827.1	2004	Argentina	RSVA		A11
KF826828.1	2004	Argentina	RSVA		A12
KF826829.1	2005	Mexico	RSVB		B6
KF826830.1	2009	Germany	RSVA		A23
KF826831.1	2009	Germany	RSVA		A23
KF826832.1	2009	Italy	RSVA		A11
KF826833.1	2009	Italy	RSVA		A23
KF826834.1	2009	Italy	RSVB		B6
KF826835.1	2009	Italy	RSVB		B6
KF826836.1	2006	Mexico	RSVA		A12
KF826837.1	2006	Mexico	RSVA		A12
KF826838.1	2006	Argentina	RSVA		A21
KF826839.1	2006	Argentina	RSVB		B6
KF826840.1	2007	Mexico	RSVA		A22
KF826841.1	2007	Argentina	RSVA		A12

KF826842.1	2007	Argentina	RSVB	B6
KF826843.1	2008	Mexico	RSVB	B6
KF826844.1	2008	Mexico	RSVB	B6
KF826845.1	2008	Argentina	RSVB	B6
KF826846.1	2008	Argentina	RSVA	A12
KF826849.1	2010	USA	RSVA	A23
KF826850.1	2008	USA	RSVA	A12
KF826851.1	2007	USA	RSVB	B6
KF826852.1	2007	USA	RSVA	A12
KF826853.2	2008	Germany	RSVB	B4
KF826854.1	2009	Italy	RSVA	A10
KF826855.1	2009	Italy	RSVA	A23
KF826856.1	2009	Italy	RSVA	A23
KF826857.1	2009	Italy	RSVB	B6
KF826858.1	2009	Italy	RSVB	B6
KF826859.1	2009	Italy	RSVB	B6
KF826860.1	2009	Italy	RSVB	B6
KF893260.1		UK	RSVB	
KJ627245.1	2010	Peru	RSVA	A23
KJ627246.1	2009	Peru	RSVA	A23
KJ627247.1	2011	Peru	RSVB	B6
KJ627248.1	2009	Peru	RSVA	A20
KJ627250.1	2010	Peru	RSVA	A23
KJ627251.1	2012	Peru	RSVB	B6
KJ627252.1	2010	Peru	RSVA	A23
KJ627253.1	2009	Peru	RSVA	A23
KJ627254.1	2011	Peru	RSVB	B6
KJ627255.1	2009	Peru	RSVA	A20
KJ627256.1	2009	Peru	RSVA	A23
KJ627257.1	2009	Peru	RSVA	A21
KJ627258.1	2010	Peru	RSVA	A23
KJ627259.1	2011	Peru	RSVA	A23
KJ627260.1	2008	Peru	RSVA	A20
KJ627261.1	2010	Peru	RSVA	A23
KJ627262.1	2012	Peru	RSVB	B6
KJ627263.1	2010	Peru	RSVA	A23
KJ627264.1	2012	Peru	RSVA	A23
KJ627266.1	2009	Peru	RSVA	A22
KJ627267.1	2010	Peru	RSVA	A23
KJ627268.1	2010	Peru	RSVA	A23
KJ627269.1	2010	Peru	RSVA	A23
KJ627270.1	2010	Peru	RSVA	A23
KJ627271.1	2009	Peru	RSVA	A23
KJ627272.1	2009	Peru	RSVA	A23
KJ627273.1	2010	Peru	RSVA	A23
KJ627274.1	2009	Peru	RSVA	A22
KJ627275.1	2010	Peru	RSVA	A23

KJ627276.1	2009	Peru	RSVA	A20
KJ627277.1	2011	Peru	RSVB	B6
KJ627278.1	2010	Peru	RSVB	B6
KJ627279.1	2010	Peru	RSVA	A23
KJ627280.1	2008	Peru	RSVB	B6
KJ627281.1	2009	Peru	RSVA	A22
KJ627282.1	2009	Peru	RSVA	A22
KJ627283.1	2010	Peru	RSVA	A23
KJ627284.1	2009	Peru	RSVA	A20
KJ627285.1	2011	Peru	RSVB	B6
KJ627286.1	2009	Peru	RSVA	A23
KJ627287.1	2010	Peru	RSVA	A23
KJ627288.1	2009	Peru	RSVA	A22
KJ627289.1	2009	Peru	RSVA	A20
KJ627290.1	2009	Peru	RSVA	A21
KJ627291.1	2009	Peru	RSVA	A23
KJ627292.1	2010	Peru	RSVA	A23
KJ627293.1	2009	Peru	RSVA	A22
KJ627294.1	2010	Peru	RSVA	A23
KJ627295.1	2010	Peru	RSVA	A23
KJ627296.1	2010	Peru	RSVA	A23
KJ627297.1	2010	Peru	RSVA	A23
KJ627298.1	2010	Peru	RSVA	A23
KJ627299.1	2007	Peru	RSVB	B6
KJ627300.1	2009	Peru	RSVA	A23
KJ627301.1	2010	Peru	RSVA	A23
KJ627302.1	2008	Peru	RSVB	B6
KJ627303.1	2010	Peru	RSVA	A23
KJ627304.1	2009	Peru	RSVA	A23
KJ627305.1	2007	Peru	RSVA	A18
KJ627306.1	2009	Peru	RSVA	A23
KJ627307.1	2010	Peru	RSVA	A23
KJ627308.1	2010	Peru	RSVA	A23
KJ627309.1	2010	Peru	RSVA	A23
KJ627310.1	2007	Peru	RSVB	B6
KJ627311.1	2008	Peru	RSVA	A20
KJ627312.1	2010	Peru	RSVA	A23
KJ627313.1	2010	Peru	RSVA	A23
KJ627314.1	2009	Peru	RSVA	A20
KJ627315.1	2009	Peru	RSVA	A23
KJ627316.1	2010	Peru	RSVA	A23
KJ627317.1	2007	Peru	RSVB	B6
KJ627318.1	2009	Peru	RSVA	A23
KJ627319.1	2009	Peru	RSVA	A23
KJ627320.1	2010	Peru	RSVA	A23
KJ627321.1	2009	Peru	RSVA	A20
KJ627322.1	2009	Peru	RSVA	A23

KJ627324.1	2009	Peru	RSVA	A20
KJ627325.1	2010	Peru	RSVA	A23
KJ627326.1	2010	Peru	RSVA	A23
KJ627327.1	2007	Peru	RSVA	A20
KJ627328.1	2010	Peru	RSVA	A23
KJ627329.1	2010	Peru	RSVA	A23
KJ627330.1	2008	Peru	RSVB	B6
KJ627332.1	2011	Peru	RSVB	B6
KJ627333.1	2010	Peru	RSVA	A23
KJ627334.1	2010	Peru	RSVA	A23
KJ627335.1	2009	Peru	RSVA	A21
KJ627336.1	2008	Peru	RSVA	A20
KJ627337.1	2009	Peru	RSVA	A21
KJ627338.1	2009	Peru	RSVA	A22
KJ627339.1	2009	Peru	RSVA	A20
KJ627340.1	2012	Peru	RSVB	B6
KJ627341.1	2011	Peru	RSVB	B6
KJ627342.1	2012	Peru	RSVB	B6
KJ627343.1	2010	Peru	RSVA	A23
KJ627344.1	2009	Peru	RSVA	A23
KJ627345.1	2010	Peru	RSVA	A23
KJ627346.1	2010	Peru	RSVA	A23
KJ627348.1	2011	Peru	RSVB	B6
KJ627349.1	2009	Peru	RSVA	A23
KJ627350.1	2009	Peru	RSVA	A23
KJ627351.1	2011	Peru	RSVA	A23
KJ627352.1	2007	Peru	RSVA	A18
KJ627353.1	2008	Peru	RSVA	A20
KJ627354.1	2009	Peru	RSVA	A22
KJ627355.1	2010	Peru	RSVA	A23
KJ627356.1	2010	Peru	RSVA	A23
KJ627357.1	2010	Peru	RSVA	A23
KJ627358.1	2010	Peru	RSVA	A23
KJ627359.1	2011	Peru	RSVB	B6
KJ627360.1	2009	Peru	RSVA	A23
KJ627361.1	2007	Peru	RSVA	A18
KJ627362.1	2009	Peru	RSVA	A22
KJ627364.1	2011	Peru	RSVB	B6
KJ627365.1	2010	Peru	RSVA	A23
KJ627366.1	2009	Peru	RSVA	A20
KJ627367.1	2010	Peru	RSVA	A23
KJ627369.1	2009	Peru	RSVA	A22
KJ627370.1	2011	Peru	RSVA	A23
KJ627371.1	2010	Peru	RSVA	A23
KJ627372.1	2010	Peru	RSVA	A23
KJ627373.1	2009	Peru	RSVA	A23
KJ627374.1	2011	Peru	RSVA	A23

KJ672424.1	2012	USA	RSVA	A23
KJ672425.1	2013	USA	RSVB	B6
KJ672426.1	2013	USA	RSVA	A23
KJ672427.1	2012	USA	RSVA	A23
KJ672428.1	2013	USA	RSVA	A23
KJ672429.1	2013	USA	RSVA	A23
KJ672430.1	2013	USA	RSVB	B6
KJ672431.1	2012	USA	RSVA	A23
KJ672432.1	2013	USA	RSVA	A23
KJ672433.1	2013	USA	RSVA	A23
KJ672434.1	2013	USA	RSVA	A23
KJ672435.1	2012	USA	RSVA	A23
KJ672436.1	2012	USA	RSVA	A23
KJ672437.1	2013	USA	RSVA	A23
KJ672438.1	2013	USA	RSVB	B6
KJ672439.1	2012	USA	RSVA	A23
KJ672440.1	2012	USA	RSVA	A23
KJ672441.1	2013	USA	RSVA	A23
KJ672442.1	2013	USA	RSVA	A23
KJ672443.1	2013	USA	RSVA	A23
KJ672444.1	2012	USA	RSVA	A23
KJ672446.1	2012	USA	RSVA	A23
KJ672447.1	2013	USA	RSVA	A12
KJ672448.1	2013	USA	RSVA	A23
KJ672449.1	2012	USA	RSVA	A23
KJ672450.1	2013	USA	RSVA	A23
KJ672451.1	2012	USA	RSVA	A23
KJ672452.1	2013	USA	RSVA	A23
KJ672453.1	2013	USA	RSVA	A23
KJ672454.1	2013	USA	RSVA	A23
KJ672455.1	2012	USA	RSVA	A23
KJ672456.1	2012	USA	RSVA	A23
KJ672457.1	2013	USA	RSVA	A23
KJ672458.1	2013	USA	RSVA	A23
KJ672459.1	2013	USA	RSVA	A23
KJ672460.1	2013	USA	RSVA	A23
KJ672461.1	2012	USA	RSVA	A23
KJ672462.1	2013	USA	RSVA	A12
KJ672463.1	2013	USA	RSVA	A23
KJ672464.1	2012	USA	RSVA	A23
KJ672465.1	2012	USA	RSVA	A23
KJ672466.1	2012	USA	RSVA	A23
KJ672467.1	2013	USA	RSVA	A23
KJ672468.1	2012	USA	RSVA	A23
KJ672469.1	2013	USA	RSVA	A23
KJ672470.1	2012	USA	RSVA	A23
KJ672471.1	2013	USA	RSVA	A23

KJ672472.1	2012	USA	RSVA	A23
KJ672473.1	2012	USA	RSVB	B6
KJ672474.1	2012	USA	RSVA	A12
KJ672475.1	2013	USA	RSVA	A23
KJ672476.1	2013	USA	RSVB	B6
KJ672477.1	2012	USA	RSVA	A23
KJ672478.1	2013	USA	RSVA	A23
KJ672479.1	2013	USA	RSVA	A12
KJ672480.1	2013	USA	RSVA	A23
KJ672481.1	2013	USA	RSVB	B6
KJ672482.1	2012	USA	RSVA	A23
KJ672483.1	2013	USA	RSVA	A12
KJ672484.1	2012	USA	RSVA	A23
KJ723460.1	1991	USA	RSVB	B3
KJ723461.1	1986	USA	RSVA	A2
KJ723462.1	1992	USA	RSVA	A7
KJ723463.2	1987	USA	RSVB	B3
KJ723464.1	1989	USA	RSVA	A14
KJ723465.1	1992	USA	RSVA	A7
KJ723466.2	1993	USA	RSVB	B2
KJ723467.1	1986	USA	RSVA	A2
KJ723468.2	1988	USA	RSVA	A3
KJ723469.1	1987	USA	RSVB	B3
KJ723470.2	1991	USA	RSVB	B3
KJ723473.1	1990	USA	RSVA	A7
KJ723474.1	1989	USA	RSVA	A2
KJ723475.1	1986	USA	RSVA	A2
KJ723476.2	1987	USA	RSVB	B3
KJ723477.1	1991	USA	RSVB	B3
KJ723478.1	1982	USA	RSVA	A2
KJ723479.1	1983	USA	RSVB	
KJ723480.2	1991	USA	RSVB	B3
KJ723481.2	1985	USA	RSVB	B3
KJ723482.2	1989	USA	RSVB	B3
KJ723483.1	1984	USA	RSVA	A13
KJ723484.2	1993	USA	RSVB	B2
KJ723485.2	1984	USA	RSVB	
KJ723487.1	1993	USA	RSVA	A7
KJ723488.1	1981	USA	RSVA	A5
KJ723489.1	1990	USA	RSVA	A2
KJ723490.1	1992	USA	RSVA	A7
KJ723491.2	1988	USA	RSVA	A2
KJ723492.1	1990	USA	RSVA	A14
KJ939919.1	2009	VietNam	RSVB	B6
KJ939920.1	2009	VietNam	RSVB	B6
KJ939921.1	2009	VietNam	RSVB	B6
KJ939922.1	2009	VietNam	RSVB	B6

KJ939923.1	2009	VietNam	RSVB	B6
KJ939924.1	2009	VietNam	RSVB	B6
KJ939925.1	2009	VietNam	RSVB	B6
KJ939926.1	2010	VietNam	RSVB	B6
KJ939927.1	2010	VietNam	RSVB	B6
KJ939928.1	2010	VietNam	RSVB	B6
KJ939929.1	2010	VietNam	RSVB	B6
KJ939930.1	2009	VietNam	RSVB	B6
KJ939931.1	2009	VietNam	RSVB	B6
KJ939932.1	2010	VietNam	RSVB	
KJ939933.1	2010	VietNam	RSVB	B6
KJ939934.1	2010	VietNam	RSVB	B6
KJ939935.1	2009	VietNam	RSVA	A23
KJ939936.1	2009	VietNam	RSVA	A23
KJ939937.1	2009	VietNam	RSVA	A23
KJ939938.1	2009	VietNam	RSVA	A23
KJ939939.1	2009	VietNam	RSVA	A23
KJ939940.1	2009	VietNam	RSVA	A23
KJ939941.1	2010	VietNam	RSVA	A23
KJ939942.1	2010	VietNam	RSVA	A23
KJ939943.1	2010	VietNam	RSVA	A10
KJ939944.1	2010	VietNam	RSVA	A23
KJ939945.1	2010	VietNam	RSVA	A23
KJ939946.1	2010	VietNam	RSVA	A23
KJ939947.1	2010	VietNam	RSVA	A23
KJ939948.1	2010	VietNam	RSVA	A10
KJ939949.1	2010	VietNam	RSVA	A23
KJ939950.1	2010	VietNam	RSVA	A23
KJ939951.1	2009	VietNam	RSVA	A23
KJ939952.1	2009	VietNam	RSVA	A23
KJ939953.1	2010	VietNam	RSVA	A23
KJ939954.1	2010	VietNam	RSVA	A10
KJ939955.1	2010	VietNam	RSVA	A23
KJ939956.1	2010	VietNam	RSVA	A23
KJ939957.1	2010	VietNam	RSVA	A23
KJ939958.1	2010	VietNam	RSVA	A23
KJ939959.1	2010	VietNam	RSVA	A23
KJ939960.1	2010	VietNam	RSVA	A23
KJ939961.1	2010	VietNam	RSVA	A23
KJ939962.1	2010	VietNam	RSVA	A23
KJ939963.1	2010	VietNam	RSVA	A23
KJ939964.1	2010	VietNam	RSVA	A23
KJ939965.1	2010	VietNam	RSVA	A23
KJ939966.1	2010	VietNam	RSVA	A23
KJ939967.1	2010	VietNam	RSVA	A23
KJ939968.1	2010	VietNam	RSVA	A23
KJ939969.1	2010	VietNam	RSVA	A23

KJ939970.1	2010	VietNam	RSVA	A23
KJ939971.1	2010	VietNam	RSVA	A23
KM042381.1	2013	USA	RSVA	A23
KM042383.1	2012	USA	RSVA	A23
KM042384.1	2013	USA	RSVA	A23
KM042385.1	2013	USA	RSVA	A23
KM042386.1	2013	USA	RSVA	A23
KM042388.1	2013	USA	RSVA	A23
KM042389.1	2012	USA	RSVA	A23
KM042390.1	2013	USA	RSVA	A23
KM042391.1	2013	USA	RSVA	A23
KM042392.1	2013	USA	RSVA	A23
KM042393.1	2012	USA	RSVB	B6
KM517572.1	2011	China	RSVA	A23
KM517573.1	2013	China	RSVB	B6
KM578843.1	2012	China	RSVA	A23
KP119745.1	2012	HongKong	RSVA	A17
KP119746.1	2012	HongKong	RSVA	A17
KP119747.1	2012	HongKong	RSVA	A17
KP119748.1	2012	HongKong	RSVA	A17
KP218910.1	2008	China	RSVA	A17
KP258695.1	1988	USA	RSVA	A2
KP258696.1	1986	USA	RSVA	A3
KP258697.1	1994	USA	RSVA	A17
KP258698.1	1991	USA	RSVB	B3
KP258699.1	1985	USA	RSVA	A6
KP258700.1	1985	USA	RSVA	A14
KP258701.1	1994	USA	RSVA	A7
KP258702.1	1994	USA	RSVB	B2
KP258703.1	1997	USA	RSVA	A9
KP258704.1	1994	USA	RSVA	A3
KP258705.1	1995	USA	RSVB	B2
KP258707.1	1995	USA	RSVA	A9
KP258708.1	1996	USA	RSVB	B4
KP258709.1	1981	USA	RSVA	A5
KP258710.1	1991	USA	RSVA	A7
KP258711.1	1992	USA	RSVA	A7
KP258712.1	1979	USA	RSVB	B3
KP258713.1	1993	USA	RSVB	B4
KP258714.1	1986	USA	RSVB	B3
KP258715.1	1988	USA	RSVA	A3
KP258717.1	1994	USA	RSVA	A2
KP258718.1	1983	USA	RSVB	
KP258719.1	1988	USA	RSVA	A2
KP258720.1	1990	USA	RSVB	B2
KP258721.1	1981	USA	RSVB	B3
KP258722.1	1993	USA	RSVA	A7

KP258723.1	1986	USA	RSVA		A13
KP258724.1	1997	USA	RSVB		B5
KP258725.1	1980	USA	RSVA		A5
KP258726.1	1996	USA	RSVA		A9
KP258727.1	1997	USA	RSVA		A10
KP258728.1	1992	USA	RSVA		A7
KP258729.1	1990	USA	RSVA		A2
KP258730.1	1987	USA	RSVA		A2
KP258731.1	1982	USA	RSVB		B3
KP258732.1	1991	USA	RSVA		A7
KP258733.1	1984	USA	RSVA		A13
KP258734.1	1989	USA	RSVA		A14
KP258735.1	1987	USA	RSVB		B3
KP258736.1	1982	USA	RSVB		
KP258737.1	1984	USA	RSVA		A15
KP258738.1	1980	USA	RSVB		
KP258739.1	1996	USA	RSVB		B4
KP258740.1	1993	USA	RSVA		A7
KP258741.1	1990	USA	RSVA		A2
KP258742.1	1994	USA	RSVB		B4
KP258743.1	1998	USA	RSVA		A15
KP258744.1	1995	USA	RSVA		A2
KP258745.1	1992	USA	RSVB		B4
KP317917.1	2006	Kenya	RSVB		B6
KP317923.1	2012	Kenya	RSVB		B4
KP317925.1	2004	Kenya	RSVB		B6
KP317927.1	2011	Kenya	RSVB		B6
KP317928.1	2011	Kenya	RSVB		B6
KP317932.1	2010	Kenya	RSVB		B6
KP317934.1	2012	Kenya	RSVB		B6
KP317941.1	2010	Kenya	RSVB		B6
KP317945.1	2011	Kenya	RSVB		B6
KP317952.1	2010	Kenya	RSVB		B6
KP317953.1	2012	Kenya	RSVA		A23
KP663728.1	2012	SouthKorea	RSVA		A23
KP663729.1	2014	SouthKorea	RSVB		B6
KP663730.1	2014	SouthKorea	RSVB		B6
KP856961.1	1989	USA	RSVB		B1
KP856963.1	1980	USA	RSVB		
KP856965.1	1989	USA	RSVB		B1
KP856966.1	1998	USA	RSVB		B4
KP856967.1	1981	USA	RSVA		A5
KP856968.1	1998	USA	RSVA		A19
KP856969.1	1979	USA	RSVA		A4
KR350475.1	2014	Mexico	RSVB		B6
KT285064.1	2014	China	RSVA	ON1	A23
KU316090.1	1981	USA	RSVA		A5

KU316091.1	1998	USA	RSVA	A19
KU316092.1	1991	USA	RSVA	A15
KU316093.1	1988	USA	RSVA	A3
KU316094.1	1989	USA	RSVB	B2
KU316095.1	1979	USA	RSVB	
KU316096.1	1996	USA	RSVA	A9
KU316097.1	1978	USA	RSVB	
KU316098.1	1984	USA	RSVA	A13
KU316099.1	1997	USA	RSVA	A2
KU316100.1	1994	USA	RSVB	B4
KU316101.1	1982	USA	RSVB	
KU316102.1	1982	USA	RSVB	B3
KU316103.1	1983	USA	RSVA	A2
KU316104.1	1991	USA	RSVA	A7
KU316105.1	1998	USA	RSVB	B4
KU316106.1	1977	USA	RSVA	A2
KU316107.1	1995	USA	RSVA	A2
KU316108.1	1987	USA	RSVB	B3
KU316109.1	1992	USA	RSVA	A2
KU316110.1	1984	USA	RSVA	A14
KU316111.1	1992	USA	RSVB	B2
KU316112.1	1980	USA	RSVA	A1
KU316113.1	1989	USA	RSVB	B3
KU316114.1	1993	USA	RSVB	B2
KU316115.1	1980	USA	RSVB	
KU316116.1	1977	USA	RSVB	
KU316117.1	1995	USA	RSVB	B2
KU316118.1	1996	USA	RSVA	A17
KU316119.1	1989	USA	RSVA	A2
KU316120.1	1985	USA	RSVA	A2
KU316121.1	1992	USA	RSVA	A7
KU316122.1	1980	USA	RSVB	
KU316123.1	1997	USA	RSVA	A2
KU316124.1	1992	USA	RSVA	A2
KU316125.1	1984	USA	RSVA	A13
KU316126.1	1984	USA	RSVA	A17
KU316127.1	1991	USA	RSVB	B3
KU316128.1	1995	USA	RSVB	B2
KU316129.1	1987	USA	RSVB	B3
KU316130.1	1985	USA	RSVB	B3
KU316131.1	1994	USA	RSVA	A17
KU316132.1	1991	USA	RSVB	B3
KU316133.1	1990	USA	RSVA	A6
KU316134.1	1994	USA	RSVB	B4
KU316135.1	1981	USA	RSVA	A5
KU316136.1	1987	USA	RSVB	
KU316137.1	1979	USA	RSVA	A4

KU316138.1	1987	USA	RSVA	A13
KU316139.1	1994	USA	RSVA	A17
KU316140.1	1978	USA	RSVA	A2
KU316141.1	1998	USA	RSVA	A19
KU316142.1	1982	USA	RSVA	A16
KU316143.1	1982	USA	RSVA	A4
KU316144.1	1991	USA	RSVB	B3
KU316145.1	1996	USA	RSVA	A9
KU316146.1	1983	USA	RSVA	A2
KU316147.1	1979	USA	RSVB	
KU316148.1	1984	USA	RSVA	A16
KU316149.1	1977	USA	RSVA	A4
KU316150.1	1978	USA	RSVA	A3
KU316151.1	1986	USA	RSVB	B3
KU316152.1	1985	USA	RSVA	A2
KU316153.1	1988	USA	RSVA	A2
KU316154.1	1985	USA	RSVA	A2
KU316155.1	1978	USA	RSVA	A2
KU316156.1	1989	USA	RSVB	B2
KU316157.1	1979	USA	RSVA	A4
KU316158.1	1996	USA	RSVB	B4
KU316159.1	1997	USA	RSVB	B2
KU316160.1	1986	USA	RSVA	A2
KU316161.1	1993	USA	RSVA	A7
KU316162.1	1988	USA	RSVA	A2
KU316163.1	1993	USA	RSVB	B2
KU316164.1	1995	USA	RSVA	A2
KU316165.1	1987	USA	RSVA	A2
KU316166.1	1977	USA	RSVA	A16
KU316167.1	1985	USA	RSVA	A14
KU316168.1	1977	USA	RSVA	A2
KU316169.1	1985	USA	RSVA	A2
KU316170.1	1995	USA	RSVA	A9
KU316171.1	1977	USA	RSVA	A1
KU316172.1	1997	USA	RSVB	B4
KU316173.1	1984	USA	RSVB	B3
KU316174.1	1987	USA	RSVA	A2
KU316175.1	1985	USA	RSVB	B3
KU316176.1	1990	USA	RSVA	A8
KU316177.1	1982	USA	RSVB	
KU316178.1	1979	USA	RSVA	A4
KU316179.1	1994	USA	RSVB	B4
KU316180.1	1998	USA	RSVA	A10
KU316181.1	1990	USA	RSVB	B3
KU316182.1	1990	USA	RSVB	B3
KU839623.1	2013	USA	RSVB	
KU839624.1	2013	USA	RSVA	A23

KU839625.1	2014	USA	RSVB	B6
KU839626.1	2013	USA	RSVA	A23
KU839627.1	2014	USA	RSVB	
KU839628.1	2013	USA	RSVB	
KU839629.1	2013	USA	RSVB	
KU839630.1	2014	USA	RSVA	A23
KU839631.1	2013	USA	RSVA	A23
KU839632.1	2013	USA	RSVB	
KU839633.1	2014	USA	RSVB	B6
KU839634.1	2013	USA	RSVB	
KU839635.1	2013	USA	RSVB	
KU839636.1	2013	USA	RSVB	B6
KU839637.1	2014	USA	RSVA	A23
KU839638.1	2013	USA	RSVB	
KU839639.1	2013	USA	RSVA	A23
KU839640.1	2013	USA	RSVB	
KU839641.1	2014	USA	RSVB	
KU950455.1	2013	USA	RSVA	A23
KU950456.1	2013	USA	RSVA	A23
KU950457.1	2005	USA	RSVB	B6
KU950458.1	2013	USA	RSVB	B6
KU950459.1	2013	USA	RSVA	A23
KU950460.1	2013	USA	RSVA	A23
KU950461.1	2013	USA	RSVB	B6
KU950462.1	2013	USA	RSVB	B6
KU950463.1	2013	USA	RSVB	B6
KU950464.1	2014	USA	RSVA	A23
KU950465.1	2013	USA	RSVB	B6
KU950466.1	2013	USA	RSVB	B6
KU950467.1	2014	USA	RSVB	B6
KU950468.1	2013	USA	RSVA	A23
KU950469.1	2012	USA	RSVA	A23
KU950470.1	2013	USA	RSVA	A23
KU950471.1	2013	USA	RSVB	B6
KU950472.1	2013	USA	RSVA	A23
KU950473.1	2012	USA	RSVA	A12
KU950474.1	2012	USA	RSVA	A23
KU950475.1	2012	USA	RSVA	A23
KU950476.1	2013	USA	RSVB	B6
KU950477.1	2014	USA	RSVB	B6
KU950478.1	2013	USA	RSVB	B6
KU950479.1	2012	USA	RSVA	A12
KU950480.1	2012	USA	RSVA	A23
KU950481.1	2013	USA	RSVB	B6
KU950482.1	2013	USA	RSVB	B6
KU950483.1	2013	USA	RSVA	A23
KU950484.1	2013	USA	RSVB	B6

KU950485.1	2012	USA	RSVA	A23
KU950486.1	2012	USA	RSVA	A23
KU950487.1	2012	USA	RSVA	A12
KU950488.1	2014	USA	RSVB	B6
KU950489.1	2013	USA	RSVB	B6
KU950491.1	2013	USA	RSVA	A23
KU950492.1	2012	USA	RSVA	A23
KU950493.1	2012	USA	RSVA	A23
KU950494.1	2013	USA	RSVB	B6
KU950495.1	2013	USA	RSVB	B6
KU950496.1	2013	USA	RSVB	B6
KU950497.1	2013	USA	RSVB	B6
KU950498.1	2012	USA	RSVA	A23
KU950499.1	2013	USA	RSVA	A23
KU950500.1	2012	USA	RSVB	B6
KU950501.1	2013	USA	RSVA	A12
KU950502.1	2012	USA	RSVA	A23
KU950503.1	2013	USA	RSVB	B6
KU950504.1	2013	USA	RSVB	B6
KU950505.1	2013	USA	RSVA	A23
KU950506.1	2013	USA	RSVA	A23
KU950507.1	2012	USA	RSVA	A23
KU950508.1	2013	USA	RSVB	B6
KU950509.1	2012	USA	RSVA	A23
KU950510.1	2013	USA	RSVB	B6
KU950511.1	2012	USA	RSVA	A23
KU950512.1	2013	USA	RSVB	B6
KU950513.1	2012	USA	RSVA	A23
KU950514.1	2013	USA	RSVB	B6
KU950515.1	2013	USA	RSVB	B6
KU950516.1	2013	USA	RSVB	
KU950517.1	2013	USA	RSVB	B6
KU950518.1	2013	USA	RSVA	A23
KU950519.1	2013	USA	RSVA	A23
KU950520.1	2013	USA	RSVA	A23
KU950521.1	2012	USA	RSVA	A23
KU950522.1	2012	USA	RSVA	A23
KU950523.1	2014	USA	RSVA	A23
KU950524.1	2012	USA	RSVA	A23
KU950525.1	2014	USA	RSVB	B6
KU950526.1	2013	USA	RSVB	B6
KU950527.1	2013	USA	RSVA	A23
KU950528.1	2013	USA	RSVA	A23
KU950529.1	2012	USA	RSVA	A23
KU950530.1	2014	USA	RSVB	B6
KU950531.1	2013	USA	RSVA	A23
KU950532.1	2013	USA	RSVB	B6

KU950533.1	2014	USA	RSVB	B6
KU950534.1	2014	USA	RSVB	B6
KU950535.1	2013	USA	RSVB	B6
KU950536.1	2012	USA	RSVA	A23
KU950537.1	2014	USA	RSVA	A23
KU950538.1	2012	USA	RSVA	A23
KU950539.1	2013	USA	RSVB	B6
KU950540.1	2013	USA	RSVA	A23
KU950541.1	2013	USA	RSVA	A23
KU950542.1	2013	USA	RSVB	B6
KU950543.1	2013	USA	RSVB	B6
KU950544.1	2013	USA	RSVA	A23
KU950545.1	2012	USA	RSVA	A23
KU950546.1	2012	USA	RSVA	A23
KU950547.1	2013	USA	RSVB	B6
KU950548.1	2014	USA	RSVB	B6
KU950549.1	2013	USA	RSVA	A23
KU950550.1	2012	USA	RSVA	A23
KU950552.1	2013	USA	RSVB	B6
KU950553.1	2013	USA	RSVB	B6
KU950554.1	2012	USA	RSVA	A23
KU950555.1	2013	USA	RSVB	B6
KU950556.1	2012	USA	RSVA	A23
KU950557.1	2012	USA	RSVA	A23
KU950558.1	2014	USA	RSVB	B6
KU950560.1	2013	USA	RSVA	A23
KU950561.1	2006	USA	RSVA	A12
KU950562.1	2013	USA	RSVB	B6
KU950563.1	2012	USA	RSVA	A23
KU950564.1	2012	USA	RSVA	A12
KU950565.1	2013	USA	RSVB	B6
KU950566.1	2013	USA	RSVA	A23
KU950567.1	2012	USA	RSVA	A23
KU950568.1	2014	USA	RSVB	B6
KU950569.1	2013	USA	RSVB	B6
KU950570.1	2012	USA	RSVA	A23
KU950571.1	2012	USA	RSVB	B6
KU950572.1	2012	USA	RSVA	A23
KU950573.1	2006	USA	RSVA	A17
KU950574.1	2005	USA	RSVB	B6
KU950576.1	2012	USA	RSVB	B6
KU950577.1	2013	USA	RSVB	B6
KU950578.1	2013	USA	RSVB	B6
KU950579.1	2012	USA	RSVA	A23
KU950580.1	2013	USA	RSVB	B6
KU950581.1	2012	USA	RSVA	A23
KU950582.1	2013	USA	RSVB	B6

KU950583.1	2013	USA	RSVA	A23
KU950584.1	2013	USA	RSVB	B6
KU950585.1	2013	USA	RSVA	A23
KU950586.1	2013	USA	RSVB	B6
KU950587.1	2013	USA	RSVB	B6
KU950588.1	2014	USA	RSVB	B6
KU950589.1	2013	USA	RSVB	B6
KU950590.1	2013	USA	RSVA	A23
KU950591.1	2013	USA	RSVB	B6
KU950592.1	2012	USA	RSVA	A23
KU950593.1	2014	USA	RSVB	B6
KU950594.1	2013	USA	RSVA	A23
KU950595.1	2012	USA	RSVA	A23
KU950596.1	2013	USA	RSVA	A23
KU950598.1	2013	USA	RSVA	A23
KU950599.1	2013	USA	RSVB	B6
KU950601.1	2013	USA	RSVB	B6
KU950602.1	2013	USA	RSVB	B6
KU950603.1	2013	USA	RSVB	B6
KU950604.1	2013	USA	RSVB	B6
KU950605.1	2013	USA	RSVB	B6
KU950606.1	2013	USA	RSVB	B6
KU950607.1	2013	USA	RSVB	B6
KU950608.1	2013	USA	RSVA	A23
KU950609.1	2012	USA	RSVA	A12
KU950610.1	2013	USA	RSVA	A23
KU950611.1	2013	USA	RSVB	B6
KU950612.1	2012	USA	RSVA	A23
KU950613.1	2013	USA	RSVB	B6
KU950614.1	2005	USA	RSVB	B6
KU950615.1	2012	USA	RSVA	A23
KU950616.1	2012	USA	RSVA	A12
KU950617.1	2012	USA	RSVA	A23
KU950618.1	2012	USA	RSVB	B6
KU950619.1	2012	USA	RSVB	B6
KU950620.1	2012	USA	RSVA	A23
KU950621.1	2014	USA	RSVB	B6
KU950622.1	2013	USA	RSVB	B6
KU950623.1	2013	USA	RSVA	A23
KU950624.1	2013	USA	RSVA	A23
KU950625.1	2014	USA	RSVB	B6
KU950626.1	2013	USA	RSVA	A23
KU950627.1	2013	USA	RSVA	A23
KU950628.1	2013	USA	RSVA	A23
KU950629.1	2013	USA	RSVA	A23
KU950630.1	2013	USA	RSVB	B6
KU950631.1	2012	USA	RSVA	A23

KU950632.1	2012	USA	RSVA	A23
KU950633.1	2013	USA	RSVB	B6
KU950634.1	2012	USA	RSVA	A23
KU950635.1	2013	USA	RSVB	B6
KU950636.1	2012	USA	RSVA	A23
KU950637.1	2014	USA	RSVB	B6
KU950638.1	2013	USA	RSVA	A23
KU950639.1	2013	USA	RSVA	A23
KU950641.1	2013	USA	RSVA	A23
KU950642.1	2012	USA	RSVA	A23
KU950643.1	2012	USA	RSVA	A23
KU950644.1	2013	USA	RSVA	A23
KU950645.1	2013	USA	RSVA	A23
KU950646.1	2012	USA	RSVA	A23
KU950647.1	2012	USA	RSVB	B6
KU950648.1	2014	USA	RSVB	B6
KU950649.1	2012	USA	RSVA	A23
KU950650.1	2013	USA	RSVA	A23
KU950651.1	2012	USA	RSVA	A23
KU950652.1	2012	USA	RSVA	A23
KU950653.1	2012	USA	RSVA	A23
KU950654.1	2012	USA	RSVA	A23
KU950655.1	2012	USA	RSVA	A23
KU950656.1	2013	USA	RSVB	B6
KU950657.1	2012	USA	RSVB	B6
KU950658.1	2012	USA	RSVA	A23
KU950659.1	2013	USA	RSVB	B6
KU950660.1	2012	USA	RSVA	A23
KU950661.1	2012	USA	RSVA	A23
KU950662.1	2012	USA	RSVB	B6
KU950663.1	2013	USA	RSVB	B6
KU950664.1	2012	USA	RSVA	A23
KU950665.1	2013	USA	RSVB	B6
KU950666.1	2013	USA	RSVA	A23
KU950667.1	2012	USA	RSVA	A23
KU950668.1	2013	USA	RSVB	B6
KU950669.1	2013	USA	RSVB	B6
KU950670.1	2012	USA	RSVA	A23
KU950671.1	2012	USA	RSVA	A23
KU950672.1	2013	USA	RSVB	B6
KU950673.1	2012	USA	RSVA	A23
KU950674.1	2012	USA	RSVA	A23
KU950675.1	2013	USA	RSVB	B6
KU950676.1	2014	USA	RSVB	B6
KU950677.1	2012	USA	RSVA	A23
KU950678.1	2013	USA	RSVB	B6
KU950679.1	2013	USA	RSVB	B6

KU950680.1	2012	USA	RSVA	A23
KU950681.1	2013	USA	RSVB	B6
KU950682.1	2012	USA	RSVB	B6
KU950683.1	2013	USA	RSVA	A23
KU950684.1	2013	USA	RSVB	
KU950685.1	2013	USA	RSVA	A23
KU950686.1	2014	USA	RSVA	A23
KU950687.1	2013	USA	RSVB	B6
KU950688.1	2013	USA	RSVB	B6
KU950689.1	2013	USA	RSVB	B6
KU950690.1	2013	USA	RSVB	B6
KU950691.1	2013	USA	RSVB	B6
KU950692.1	2012	USA	RSVA	A23
KU950694.1	2012	USA	RSVA	A23
KU950695.1	2013	USA	RSVB	
KU950696.1	2012	USA	RSVA	A23
KU950697.1	2013	USA	RSVB	B6
KU950698.1	2013	USA	RSVA	A23
KX765886.1	2015	NewZealand	RSVA	A23
KX765887.1	2015	NewZealand	RSVA	A23
KX765888.1	2014	NewZealand	RSVB	B6
KX765889.1	2012	NewZealand	RSVB	B6
KX765890.1	2011	NewZealand	RSVB	B6
KX765891.1	2013	NewZealand	RSVA	A12
KX765892.1	2014	NewZealand	RSVB	B6
KX765893.1	2010	NewZealand	RSVB	B6
KX765894.1	2011	NewZealand	RSVA	A23
KX765895.1	2014	NewZealand	RSVB	B6
KX765896.1	2011	NewZealand	RSVA	A23
KX765897.1	2013	NewZealand	RSVB	B6
KX765898.1	2015	NewZealand	RSVA	A23
KX765899.1	2010	NewZealand	RSVB	B6
KX765900.1	2010	NewZealand	RSVB	B6
KX765901.1	2012	NewZealand	RSVA	A23
KX765902.1	2014	NewZealand	RSVA	A23
KX765903.1	2011	NewZealand	RSVB	B6
KX765904.1	2013	NewZealand	RSVA	A23
KX765905.1	2014	NewZealand	RSVB	B6
KX765906.1	2015	NewZealand	RSVB	B6
KX765907.1	2014	NewZealand	RSVB	B6
KX765908.1	2013	NewZealand	RSVB	B6
KX765909.1	2013	NewZealand	RSVB	B6
KX765910.1	2011	NewZealand	RSVA	A23
KX765911.1	2011	NewZealand	RSVA	A23
KX765913.1	2012	NewZealand	RSVA	A23
KX765914.1	2015	NewZealand	RSVA	A23
KX765915.1	2014	NewZealand	RSVA	A23

KX765916.1	2015	NewZealand	RSVA	A23
KX765917.1	2014	NewZealand	RSVA	A23
KX765918.1	2011	NewZealand	RSVA	A23
KX765919.1	2013	NewZealand	RSVA	A23
KX765920.1	2012	NewZealand	RSVA	A23
KX765921.1	2014	NewZealand	RSVB	B6
KX765922.1	2011	NewZealand	RSVB	B6
KX765923.1	2013	NewZealand	RSVB	B6
KX765925.1	2015	NewZealand	RSVA	A23
KX765926.1	2012	NewZealand	RSVA	A23
KX765927.1	2011	NewZealand	RSVB	B6
KX765928.1	2011	NewZealand	RSVA	A23
KX765929.1	2014	NewZealand	RSVB	B6
KX765930.1	2010	NewZealand	RSVB	B6
KX765931.1	2011	NewZealand	RSVA	A23
KX765932.1	2015	NewZealand	RSVA	A23
KX765933.1	2012	NewZealand	RSVA	A11
KX765934.1	2012	NewZealand	RSVA	A23
KX765935.1	2015	NewZealand	RSVB	B6
KX765936.1	2012	NewZealand	RSVA	A23
KX765937.1	2012	NewZealand	RSVA	A23
KX765938.1	2011	NewZealand	RSVA	A23
KX765939.1	2013	NewZealand	RSVA	A23
KX765940.1	2013	NewZealand	RSVA	A23
KX765941.1	2015	NewZealand	RSVA	A23
KX765942.1	2011	NewZealand	RSVA	A23
KX765943.1	2013	NewZealand	RSVB	B6
KX765944.1	2015	NewZealand	RSVA	A23
KX765945.1	2010	NewZealand	RSVB	B6
KX765946.1	2012	NewZealand	RSVA	A23
KX765947.1	2014	NewZealand	RSVB	B6
KX765948.1	2015	NewZealand	RSVA	A23
KX765949.1	2011	NewZealand	RSVB	B6
KX765950.1	2015	NewZealand	RSVA	A23
KX765951.1	2015	NewZealand	RSVA	A23
KX765952.1	2012	NewZealand	RSVB	B6
KX765953.1	2012	NewZealand	RSVA	A23
KX765954.1	2013	NewZealand	RSVA	A23
KX765955.1	2015	NewZealand	RSVA	A23
KX765956.1	2015	NewZealand	RSVA	A23
KX765957.1	2014	NewZealand	RSVB	B6
KX765958.1	2012	NewZealand	RSVA	A23
KX765959.1	2013	NewZealand	RSVB	B6
KX765960.1	2011	NewZealand	RSVA	A23
KX765961.1	2015	NewZealand	RSVB	B6
KX765962.1	2013	NewZealand	RSVB	B6
KX765963.1	2010	NewZealand	RSVB	B6

KX765964.1	2012	NewZealand	RSVB		B6
KX765965.1	2013	NewZealand	RSVB		B6
KX765966.1	2014	NewZealand	RSVB		B6
KX765967.1	2012	NewZealand	RSVA		A23
KX765968.1	2010	NewZealand	RSVB		B6
KX765969.1	2012	NewZealand	RSVA		A23
KX765970.1	2014	NewZealand	RSVA		A23
KX765971.1	2013	NewZealand	RSVA		A23
KX765972.1	2013	NewZealand	RSVA		A23
KX765973.1	2014	NewZealand	RSVB		B6
KX765974.1	2013	NewZealand	RSVA		A23
KX765975.2	2011	NewZealand	RSVB		B6
KX765976.1	2012	NewZealand	RSVB		B6
KX765977.1	2014	NewZealand	RSVA		A23
KX765978.1	2011	NewZealand	RSVB		B6
KX894796.1	2012	USA	RSVA		A23
KX894797.1	2013	USA	RSVA		A23
KX894798.1	2013	USA	RSVA		A12
KX894799.1	2012	USA	RSVA		A23
KX894800.1	2013	USA	RSVA		A12
KX894802.1	2012	USA	RSVA		A23
KX894803.1	2013	USA	RSVA		A23
KX894804.1	2013	USA	RSVA		A23
KX894805.1	2013	USA	RSVA		A23
KX894806.1	2013	USA	RSVA		A23
KX894807.1	2013	USA	RSVA		A23
KY249656.1	2014	UnitedKingdom	RSVB	BA10	B6
KY249657.1	2014	UnitedKingdom	RSVB	BA10	B6
KY249658.1	2013	UnitedKingdom	RSVB	BA10	B6
KY249659.1	2013	UnitedKingdom	RSVB	BA9	B6
KY249660.1	2013	UnitedKingdom	RSVB	BA10	B6
KY249661.1	2013	UnitedKingdom	RSVB	BA9	B6
KY249662.1	2013	UnitedKingdom	RSVB	BA9	B6
KY249663.1	2013	UnitedKingdom	RSVB	BA10	B6
KY249664.1	2013	UnitedKingdom	RSVB	BA10	B6
KY249665.1	2013	UnitedKingdom	RSVB	BA10	B6
KY249666.1	2013	UnitedKingdom	RSVB	BA9	B6
KY249667.1	2012	UnitedKingdom	RSVB	BA10	B6
KY249668.1	2012	UnitedKingdom	RSVB	BA10	B6
KY249669.1	2012	UnitedKingdom	RSVB	BA10	B6
KY249670.1	2012	UnitedKingdom	RSVB	BA10	B6
KY249671.1	2012	UnitedKingdom	RSVB	BA10	B6
KY249672.1	2012	UnitedKingdom	RSVB	BA10	B6
KY249673.1	2012	UnitedKingdom	RSVB	BA10	B6
KY249674.1	2012	UnitedKingdom	RSVB	BA9	B6
KY249675.1	2012	UnitedKingdom	RSVB	BA10	B6
KY249676.1	2012	UnitedKingdom	RSVB	BA10	B6

KY249677.1	2012	UnitedKingdom	RSVB	BA10	B6
KY249678.1	2011	UnitedKingdom	RSVB	BA10	B6
KY249679.1	2011	UnitedKingdom	RSVB	BA10	B6
KY249680.1	2011	UnitedKingdom	RSVB	BA10	B6
KY249681.1	2016	UnitedKingdom	RSVB	BA10	B6
KY249682.1	2016	UnitedKingdom	RSVB	BA10	B6
KY249683.1	2016	UnitedKingdom	RSVB	BA10	B6
KY418147.1	2006	USA	RSVA	GA1	
KY418148.1	2006	USA	RSVA	GA1	
KY460517.1	2010	Taiwan	RSVA		A23
KY654506.1	2012	Philippines	RSVA	NA1	A23
KY654507.1	2012	Philippines	RSVA	NA1	A23
KY654508.1	2012	Philippines	RSVA	NA1	A23
KY654509.1	2012	Philippines	RSVA	NA1	A23
KY654510.1	2012	Philippines	RSVA	NA1	A23
KY654511.1	2013	Philippines	RSVA	NA1	A23
KY654512.1	2012	Philippines	RSVA	ON1	A23
KY654513.1	2013	Philippines	RSVA	ON1	A23
KY654514.1	2013	Philippines	RSVA	ON1	A23
KY654515.1	2012	Philippines	RSVA	ON1	A23
KY654516.1	2013	Philippines	RSVA	ON1	A23
KY654517.1	2013	Philippines	RSVA	ON1	A23
KY654518.1	2013	Philippines	RSVA	ON1	A23
KY674983.1	2016	USA	RSVB		B3
KY674984.1	2016	USA	RSVB		
KY684758.1	2016	USA	RSVB		
KY782635.1	2009	China	RSVA		A23
KY883566.2	2015	Argentina	RSVA		A23
KY883567.1	2015	Argentina	RSVA		
KY883568.2	2015	Argentina	RSVB		B6
KY883569.1	2015	Argentina	RSVB		B6
KY883570.2	2015	Argentina	RSVB		B6
KY883571.2	2015	Argentina	RSVB		B6
KY924878.1	2016	China	RSVB	BA9	
KY967362.1	2015	USA	RSVA		A23
KY967363.1	2015	USA	RSVA		A23
KY967364.1	2015	USA	RSVA		A10
KY982516.1	2013	USA	RSVA		A23
KY982517.1	2013	USA	RSVA		A23
LC384997.1	2014	Philippines	RSVB		B6
LC384998.1	2015	Philippines	RSVB		B6
LC384999.1	2014	Philippines	RSVB		B6
LC385000.1	2015	Philippines	RSVB		B6
LC385001.1	2014	Philippines	RSVB		B6
LC385002.1	2015	Philippines	RSVB		B6
LC385005.2	2014	Philippines	RSVB		B6
LC385006.1	2016	Philippines	RSVB		B6

LC385007.1	2015	Philippines	RSVB		B6
LC385008.1	2016	Philippines	RSVB		B6
LC474522.1	2010	Japan	RSVB		B6
LC474523.1	2010	Japan	RSVB		B6
LC474524.1	2010	Japan	RSVB		
LC474525.1	2014	Japan	RSVB		B6
LC474526.1	2011	Japan	RSVB		B6
LC474527.1	2011	Japan	RSVB		B6
LC474528.1	2011	Japan	RSVB		B6
LC474529.1	2011	Japan	RSVB		B6
LC474530.1	2011	Japan	RSVB		B6
LC474531.1	2013	Japan	RSVB		
LC474532.1	2013	Japan	RSVB		
LC474533.1	2013	Japan	RSVB		
LC474534.1	2013	Japan	RSVB		
LC474535.1	2014	Japan	RSVB		B6
LC474536.1	2015	Japan	RSVB		
LC474537.1	2015	Japan	RSVB		
LC474538.1	2015	Japan	RSVB		
LC474539.1	2015	Japan	RSVB		
LC474540.1	2015	Japan	RSVB		
LC474541.1	2015	Japan	RSVB		
LC474542.1	2016	Japan	RSVB		
LC474543.1	2016	Japan	RSVB		
LC474544.1	2016	Japan	RSVB		
LC474545.1	2016	Japan	RSVB		
LC474546.1	2016	Japan	RSVB		
LC474547.1	2016	Japan	RSVB		
LC474548.1	2016	Japan	RSVB		
LC474549.1	2016	Japan	RSVB		
LC474550.1	2016	Japan	RSVB		
LC474551.1	2016	Japan	RSVB		
LC474552.1	2017	Japan	RSVB		
LC474553.1	2017	Japan	RSVB		
LC474554.1	2017	Japan	RSVB		
LC474555.1	2017	Japan	RSVB		
LC474556.1	2014	USA	RSVA		A23
LC474557.1	2014	USA	RSVA		A23
LC474558.1	2014	USA	RSVA		A23
LC474559.1	2014	USA	RSVB		
LC474560.1	2014	USA	RSVB		
LC488177.1	2016	Japan	RSVB	BA9	
LC495297.1	2018	Japan	RSVB		
LC530050.1	2019	Japan	RSVA		A23
MF001038.1	2015	USA	RSVA		A11
MF001039.1	2015	USA	RSVA		A23
MF001040.1	2015	USA	RSVB		B6

MF001041.1	2015	USA	RSVA	A23
MF001042.1	2015	USA	RSVB	B6
MF001043.1	2015	USA	RSVA	A23
MF001044.1	2015	USA	RSVB	B6
MF001045.1	2015	USA	RSVB	B6
MF001046.1	2015	USA	RSVB	B6
MF001047.1	2015	USA	RSVA	A22
MF001048.1	2015	USA	RSVB	
MF001049.1	2015	USA	RSVA	A23
MF001050.1	2015	USA	RSVA	A23
MF001051.1	2015	USA	RSVA	A23
MF001052.1	2015	USA	RSVA	A23
MF001053.1	2015	USA	RSVA	A23
MF001054.1	2015	USA	RSVA	A23
MF001055.1	2015	USA	RSVB	B6
MF001056.1	2015	USA	RSVA	A23
MF001057.1	2015	USA	RSVA	A23
MF001058.1	2015	USA	RSVB	
MF614946.1	2012	China	RSVA	A23
MF614947.1	2013	China	RSVA	A23
MF973154.1	?	USA	RSVB	B6
MF973155.1	?	USA	RSVB	B6
MG027862.1	2003	USA	RSVA	A11
MG642024.1	1981	USA	RSVA	A5
MG642025.1	1982	USA	RSVB	
MG642026.1	1982	USA	RSVA	A16
MG642027.1	1991	USA	RSVB	B3
MG642028.1	1980	USA	RSVA	A5
MG642030.1	1988	USA	RSVA	A15
MG642031.1	1982	USA	RSVA	A6
MG642032.1	1980	USA	RSVA	A5
MG642033.1	1994	USA	RSVA	A17
MG642034.1	1985	USA	RSVA	A15
MG642035.1	1984	USA	RSVA	A13
MG642036.1	1989	USA	RSVB	B3
MG642037.1	1980	USA	RSVB	
MG642038.1	1985	USA	RSVA	A2
MG642039.1	1985	USA	RSVB	B3
MG642040.1	1980	USA	RSVA	A5
MG642042.1	1985	USA	RSVB	B3
MG642043.1	1982	USA	RSVB	B3
MG642044.1	1982	USA	RSVB	
MG642045.1	1979	USA	RSVB	
MG642046.1	1984	USA	RSVB	B3
MG642047.1	1980	USA	RSVB	
MG642048.1	1990	USA	RSVA	A8
MG642049.1	1993	USA	RSVB	B2

MG642050.1	1994	USA	RSVA		A17
MG642051.1	1986	USA	RSVB		B3
MG642052.1	1994	USA	RSVA		A7
MG642054.1	1991	USA	RSVB		B3
MG642055.1	1990	USA	RSVA		A8
MG642056.1	1982	USA	RSVA		A16
MG642057.1	1984	USA	RSVB		B3
MG642058.1	1980	USA	RSVA		A5
MG642059.1	1983	USA	RSVB		
MG642060.1	1980	USA	RSVA		A1
MG642061.1	1994	USA	RSVA		A7
MG642062.1	1996	USA	RSVB		B2
MG642063.1	1982	USA	RSVA		A14
MG642064.1	1995	USA	RSVB		B2
MG642065.1	1981	USA	RSVB		
MG642066.1	1990	USA	RSVB		B3
MG642067.1	1987	USA	RSVA		A2
MG642068.1	1987	USA	RSVB		B3
MG642069.1	1985	USA	RSVA		A2
MG642070.1	1986	USA	RSVA		A13
MG642071.1	1984	USA	RSVA		A13
MG642072.1	1991	USA	RSVB		B3
MG642074.1	1980	USA	RSVA		A4
MG642075.1	1986	USA	RSVA		A2
MG642076.1	1987	USA	RSVB		B3
MG642077.1	1987	USA	RSVA		A13
MG642078.1	1982	USA	RSVB		
MG642079.1	1982	USA	RSVA		A6
MG642080.1	1994	USA	RSVA		A17
MG642081.1	1982	USA	RSVA		A16
MG642082.1	1989	USA	RSVB		B3
MG642083.1	1987	USA	RSVA		A6
MG793382.1	2015	Lebanon	RSVA		A23
MG813977.1	2013	USA	RSVA	ON1	A23
MG813978.1	2012	USA	RSVA	ON1	A23
MG813979.1	2012	USA	RSVA	ON1	A23
MG813980.1	2012	USA	RSVA	ON1	A23
MG813981.1	2012	USA	RSVA	ON1	A23
MG813982.1	2012	USA	RSVA	ON1	A23
MG813983.1	2012	USA	RSVA	ON1	A23
MG813984.1	2014	USA	RSVA	ON1	A23
MG813985.1	2014	USA	RSVA	ON1	A23
MG813986.1	2015	USA	RSVA	ON1	A23
MG813987.1	2015	USA	RSVA	ON1	A23
MG813988.1	2012	USA	RSVA	ON1	A23
MG813989.1	1989	USA	RSVA	ON1	A23
MG813990.1	2012	USA	RSVA	ON1	A23

MG813991.1	2012	USA	RSVA	ON1	A23
MG813992.1	2013	USA	RSVA	ON1	A23
MG813993.1	2013	USA	RSVA	ON1	A23
MG813994.1	2010	USA	RSVB	BA9	B6
MG813995.1	1962	USA	RSVB	GB1	
MG839543.1	2015	Argentina	RSVA		A23
MG839545.1	2016	Argentina	RSVB		B6
MG839547.1	2016	Argentina	RSVB		B6
MH279547.1	2017	China	RSVA	ON1	A23
MH290724.1	2017	China	RSVA	ON1	A23
MH327947.1	2017	India	RSVB		
MH383066.1	2014	Lebanon	RSVA		A23
MH447951.1	2011	Thailand	RSVA		A23
MH447952.1	2012	Thailand	RSVA		A23
MH447953.1	2013	Thailand	RSVA		A23
MH447954.1	2013	Thailand	RSVA		A23
MH447955.1	2014	Thailand	RSVA		A23
MH447956.1	2014	Thailand	RSVA		A23
MH447957.1	2016	Thailand	RSVA		A23
MH447958.1	2016	Thailand	RSVA		A23
MH447959.1	2017	Thailand	RSVA		A23
MH447960.1	2017	Thailand	RSVA		A23
MH685715.1	2013	Uganda	RSVB		B6
MK109763.1	2011	Jordan	RSVA		A23
MK109764.1	2013	Jordan	RSVB		B6
MK109765.1	2011	Jordan	RSVB		B6
MK109766.1	2013	Jordan	RSVB		B6
MK109767.1	2013	Jordan	RSVB		B6
MK109768.1	2013	Jordan	RSVB		B6
MK109769.1	2011	Jordan	RSVB		B6
MK109770.1	2010	Jordan	RSVB		B6
MK109771.1	2013	Jordan	RSVB		B6
MK109772.1	2011	Jordan	RSVA		A23
MK109773.1	2011	Jordan	RSVA		A23
MK109774.1	2011	Jordan	RSVA		A23
MK109775.1	2011	Jordan	RSVA		A23
MK109776.1	2011	Jordan	RSVA		A23
MK109777.1	2011	Jordan	RSVA		A23
MK109778.1	2012	Jordan	RSVB		B6
MK109779.1	2010	Jordan	RSVB		B6
MK109780.1	2013	Jordan	RSVB		B6
MK109781.1	2011	Jordan	RSVB		B6
MK109782.1	2013	Jordan	RSVB		B6
MK109783.1	2013	Jordan	RSVB		B6
MK109784.1	2013	Jordan	RSVB		B6
MK109785.1	2010	Jordan	RSVA		A23
MK109786.1	2012	Jordan	RSVB		B6

MK109787.1	2011	Jordan	RSVA	A23
MK109788.1	2013	Jordan	RSVB	B6
MK109789.1	2011	Jordan	RSVB	B6
MK167034.1	2005	USA	RSVB	B6
MK167035.1	2008	USA	RSVA	A23
MK453523.1	2009	USA	RSVB	B6
MK481079.1	2018	Russia	RSVB	
MK534510.1	2018	Russia	RSVB	
MK534511.1	2018	Russia	RSVB	
MK534512.1	2018	Russia	RSVB	
MK749866.1	2015	Nicaragua	RSVB	
MK749867.1	2016	Nicaragua	RSVA	A23
MK749868.1	2016	Nicaragua	RSVB	
MK749869.1	2016	Nicaragua	RSVB	
MK749870.1	2015	Nicaragua	RSVB	
MK749871.1	2015	Nicaragua	RSVB	
MK749872.1	2016	Nicaragua	RSVB	
MK749873.1	2015	Nicaragua	RSVB	
MK749874.1	2015	Nicaragua	RSVB	
MK749875.1	2016	Nicaragua	RSVB	
MK749876.1	2015	Nicaragua	RSVB	
MK749877.1	2015	Nicaragua	RSVB	
MK749878.1	2015	Nicaragua	RSVB	
MK749879.1	2015	Nicaragua	RSVB	
MK749880.1	2015	Nicaragua	RSVB	
MK749881.1	2015	Nicaragua	RSVB	
MK749882.1	2016	Nicaragua	RSVB	
MK749883.1	2015	Nicaragua	RSVB	B6
MK749884.1	2016	Nicaragua	RSVA	A23
MK749885.1	2016	Nicaragua	RSVB	
MK749886.1	2016	Nicaragua	RSVA	A23
MK749887.1	2015	Nicaragua	RSVB	
MK749888.1	2015	Nicaragua	RSVB	
MK749889.1	2015	Nicaragua	RSVB	
MK749890.1	2016	Nicaragua	RSVA	A23
MK749891.1	2015	Nicaragua	RSVB	
MK749892.1	2015	Nicaragua	RSVB	
MK749893.1	2016	Nicaragua	RSVA	A23
MK749894.1	2016	Nicaragua	RSVB	
MK749895.1	2016	Nicaragua	RSVA	A23
MK749896.1	2015	Nicaragua	RSVB	
MK749897.1	2016	Nicaragua	RSVB	
MK749898.1	2015	Nicaragua	RSVB	
MK749899.1	2016	Nicaragua	RSVB	
MK749900.1	2013	Nicaragua	RSVB	
MK749901.1	2016	Nicaragua	RSVB	
MK749902.1	2015	Nicaragua	RSVB	

MK749903.1	2015	Nicaragua	RSVB	
MK749904.1	2015	Nicaragua	RSVB	
MK749905.1	2015	Nicaragua	RSVB	
MK749906.1	2015	Nicaragua	RSVB	
MK749907.1	2015	Nicaragua	RSVB	
MK749908.1	2016	Nicaragua	RSVB	
MK749909.1	2016	Nicaragua	RSVA	A23
MK749910.1	2016	Nicaragua	RSVB	
MK749911.1	2016	Nicaragua	RSVA	A23
MK749912.1	2015	Nicaragua	RSVA	A23
MK749913.1	2016	Nicaragua	RSVA	A23
MK749914.1	2015	Nicaragua	RSVB	
MK749915.1	2016	Nicaragua	RSVB	
MK749916.1	2016	Nicaragua	RSVB	
MK749917.1	2016	Nicaragua	RSVA	A23
MN163125.1	2017	China	RSVB	
MN163126.1	2017	China	RSVB	
MN251607.1	2011	USA	RSVA	A23
MN306017.1	2018	USA	RSVA	A23
MN306029.1	2019	USA	RSVA	A23
MN306030.1	2019	USA	RSVA	A23
MN306031.1	2019	USA	RSVA	A23
MN306044.1	2019	USA	RSVB	
MN306045.1	2019	USA	RSVA	A23
MN306048.1	2019	USA	RSVA	A23
MN306050.1	2019	USA	RSVA	A23
MN306054.1	2019	USA	RSVA	A23
MN310477.1	2018	USA	RSVA	A23
MN365304.1	2016	Kenya	RSVB	
MN365305.1	2016	Kenya	RSVB	
MN365306.1	2016	Kenya	RSVB	
MN365307.1	2016	Kenya	RSVB	
MN365308.1	2016	Kenya	RSVB	
MN365310.1	2016	Kenya	RSVB	
MN365311.1	2016	Kenya	RSVB	
MN365312.1	2016	Kenya	RSVB	
MN365313.1	2016	Kenya	RSVB	
MN365314.1	2016	Kenya	RSVB	
MN365315.1	2016	Kenya	RSVB	
MN365316.1	2016	Kenya	RSVB	
MN365318.1	2017	Kenya	RSVB	
MN365319.1	2017	Kenya	RSVB	
MN365320.1	2017	Kenya	RSVB	
MN365321.1	2017	Kenya	RSVB	
MN365322.1	2017	Kenya	RSVB	
MN365323.1	2017	Kenya	RSVB	
MN365324.1	2017	Kenya	RSVB	

MN365325.1	2017	Kenya	RSVB
MN365326.1	2017	Kenya	RSVB
MN365327.1	2017	Kenya	RSVB
MN365328.1	2017	Kenya	RSVB
MN365329.1	2017	Kenya	RSVB
MN365330.1	2017	Kenya	RSVB
MN365331.1	2017	Kenya	RSVB
MN365332.1	2017	Kenya	RSVB
MN365333.1	2017	Kenya	RSVB
MN365334.1	2017	Kenya	RSVB
MN365335.1	2017	Kenya	RSVB
MN365336.1	2017	Kenya	RSVB
MN365337.1	2017	Kenya	RSVB
MN365338.1	2015	Kenya	RSVB
MN365339.1	2016	Kenya	RSVB
MN365340.1	2016	Kenya	RSVB
MN365341.1	2016	Kenya	RSVB
MN365342.1	2016	Kenya	RSVB
MN365343.1	2016	Kenya	RSVB
MN365344.1	2017	Kenya	RSVB
MN365346.1	2017	Kenya	RSVB
MN365347.1	2017	Kenya	RSVB
MN365348.1	2017	Kenya	RSVB
MN365349.1	2017	Kenya	RSVB
MN365350.1	2017	Kenya	RSVB
MN365351.1	2017	Kenya	RSVB
MN365352.1	2017	Kenya	RSVB
MN365353.1	2017	Kenya	RSVB
MN365354.1	2017	Kenya	RSVB
MN365355.1	2017	Kenya	RSVB
MN365356.1	2017	Kenya	RSVB
MN365357.1	2017	Kenya	RSVB
MN365358.1	2017	Kenya	RSVB
MN365359.1	2017	Kenya	RSVB
MN365360.1	2017	Kenya	RSVB
MN365361.1	2017	Kenya	RSVB
MN365362.1	2017	Kenya	RSVB
MN365363.1	2017	Kenya	RSVB
MN365364.1	2015	Kenya	RSVB
MN365365.1	2015	Kenya	RSVB
MN365366.1	2015	Kenya	RSVB
MN365367.1	2016	Kenya	RSVB
MN365368.1	2016	Kenya	RSVB
MN365369.1	2016	Kenya	RSVB
MN365370.1	2016	Kenya	RSVB
MN365371.1	2016	Kenya	RSVB
MN365372.1	2017	Kenya	RSVB

MN365373.1	2017	Kenya	RSVB
MN365374.1	2017	Kenya	RSVB
MN365375.1	2017	Kenya	RSVB
MN365376.1	2017	Kenya	RSVB
MN365377.1	2017	Kenya	RSVB
MN365378.1	2017	Kenya	RSVB
MN365379.1	2017	Kenya	RSVB
MN365380.1	2017	Kenya	RSVB
MN365381.1	2017	Kenya	RSVB
MN365382.1	2017	Kenya	RSVB
MN365383.1	2017	Kenya	RSVB
MN365384.1	2017	Kenya	RSVB
MN365385.1	2017	Kenya	RSVB
MN365386.1	2017	Kenya	RSVB
MN365387.1	2017	Kenya	RSVB
MN365388.1	2017	Kenya	RSVB
MN365389.1	2017	Kenya	RSVB
MN365390.1	2016	Kenya	RSVB
MN365391.1	2016	Kenya	RSVB
MN365393.1	2016	Kenya	RSVB
MN365395.1	2016	Kenya	RSVB
MN365396.1	2016	Kenya	RSVB
MN365397.1	2016	Kenya	RSVB
MN365398.1	2016	Kenya	RSVB
MN365399.1	2016	Kenya	RSVB
MN365400.1	2016	Kenya	RSVB
MN365401.1	2016	Kenya	RSVB
MN365402.1	2016	Kenya	RSVB
MN365404.1	2016	Kenya	RSVB
MN365405.1	2016	Kenya	RSVB
MN365406.1	2016	Kenya	RSVB
MN365407.1	2016	Kenya	RSVB
MN365409.1	2017	Kenya	RSVB
MN365410.1	2017	Kenya	RSVB
MN365411.1	2017	Kenya	RSVB
MN365412.1	2017	Kenya	RSVB
MN365413.1	2017	Kenya	RSVB
MN365414.1	2017	Kenya	RSVB
MN365415.1	2017	Kenya	RSVB
MN365416.1	2017	Kenya	RSVB
MN365417.1	2017	Kenya	RSVB
MN365418.1	2017	Kenya	RSVB
MN365419.1	2017	Kenya	RSVB
MN365420.1	2017	Kenya	RSVB
MN365421.1	2015	Kenya	RSVB
MN365422.1	2016	Kenya	RSVB
MN365424.1	2016	Kenya	RSVB

MN365427.1	2016	Kenya	RSVB
MN365430.1	2016	Kenya	RSVB
MN365434.1	2016	Kenya	RSVB
MN365435.1	2016	Kenya	RSVB
MN365437.1	2016	Kenya	RSVB
MN365438.1	2016	Kenya	RSVB
MN365439.1	2016	Kenya	RSVB
MN365440.1	2017	Kenya	RSVB
MN365441.1	2017	Kenya	RSVB
MN365442.1	2017	Kenya	RSVB
MN365443.1	2017	Kenya	RSVB
MN365444.1	2017	Kenya	RSVB
MN365445.1	2017	Kenya	RSVB
MN365446.1	2017	Kenya	RSVB
MN365447.1	2017	Kenya	RSVB
MN365448.1	2017	Kenya	RSVB
MN365450.1	2017	Kenya	RSVB
MN365451.1	2017	Kenya	RSVB
MN365452.1	2017	Kenya	RSVB
MN365453.1	2017	Kenya	RSVB
MN365454.1	2017	Kenya	RSVB
MN365455.1	2017	Kenya	RSVB
MN365456.1	2017	Kenya	RSVB
MN365457.1	2017	Kenya	RSVB
MN365458.1	2017	Kenya	RSVB
MN365459.1	2017	Kenya	RSVB
MN365460.1	2017	Kenya	RSVB
MN365461.1	2017	Kenya	RSVB
MN365462.1	2017	Kenya	RSVB
MN365463.1	2017	Kenya	RSVB
MN365464.1	2017	Kenya	RSVB
MN365465.1	2017	Kenya	RSVB
MN365466.1	2017	Kenya	RSVB
MN365467.1	2017	Kenya	RSVB
MN365468.1	2017	Kenya	RSVB
MN365469.1	2017	Kenya	RSVB
MN365470.1	2017	Kenya	RSVB
MN365471.1	2017	Kenya	RSVB
MN365472.1	2017	Kenya	RSVB
MN365473.1	2017	Kenya	RSVB
MN365474.1	2017	Kenya	RSVB
MN365475.1	2016	Kenya	RSVB
MN365476.1	2016	Kenya	RSVB
MN365477.1	2016	Kenya	RSVB
MN365478.1	2016	Kenya	RSVB
MN365480.1	2016	Kenya	RSVB
MN365485.1	2016	Kenya	RSVB

MN365486.1	2016	Kenya	RSVB
MN365487.1	2017	Kenya	RSVB
MN365488.1	2017	Kenya	RSVB
MN365489.1	2017	Kenya	RSVB
MN365490.1	2017	Kenya	RSVB
MN365491.1	2017	Kenya	RSVB
MN365492.1	2017	Kenya	RSVB
MN365493.1	2017	Kenya	RSVB
MN365494.1	2017	Kenya	RSVB
MN365495.1	2017	Kenya	RSVB
MN365496.1	2017	Kenya	RSVB
MN365497.1	2017	Kenya	RSVB
MN365498.1	2017	Kenya	RSVB
MN365499.1	2017	Kenya	RSVB
MN365500.1	2017	Kenya	RSVB
MN365501.1	2017	Kenya	RSVB
MN365502.1	2017	Kenya	RSVB
MN365503.1	2017	Kenya	RSVB
MN365504.1	2017	Kenya	RSVB
MN365505.1	2017	Kenya	RSVB
MN365506.1	2016	Kenya	RSVB
MN365507.1	2016	Kenya	RSVB
MN365509.1	2016	Kenya	RSVB
MN365510.1	2016	Kenya	RSVB
MN365512.1	2016	Kenya	RSVB
MN365514.1	2016	Kenya	RSVB
MN365515.1	2017	Kenya	RSVB
MN365516.1	2017	Kenya	RSVB
MN365517.1	2017	Kenya	RSVB
MN365518.1	2017	Kenya	RSVB
MN365519.1	2017	Kenya	RSVB
MN365520.1	2017	Kenya	RSVB
MN365521.1	2017	Kenya	RSVB
MN365522.1	2017	Kenya	RSVB
MN365523.1	2017	Kenya	RSVB
MN365524.1	2017	Kenya	RSVB
MN365526.1	2017	Kenya	RSVB
MN365527.1	2017	Kenya	RSVB
MN365528.1	2017	Kenya	RSVB
MN365529.1	2017	Kenya	RSVB
MN365530.1	2017	Kenya	RSVB
MN365531.1	2017	Kenya	RSVB
MN365532.1	2017	Kenya	RSVB
MN365533.1	2017	Kenya	RSVB
MN365534.1	2017	Kenya	RSVB
MN365535.1	2017	Kenya	RSVB
MN365536.1	2016	Kenya	RSVB

MN365537.1	2016	Kenya	RSVB	
MN365538.1	2016	Kenya	RSVB	
MN365539.1	2016	Kenya	RSVB	
MN365540.1	2016	Kenya	RSVB	
MN365541.1	2016	Kenya	RSVB	
MN365542.1	2016	Kenya	RSVB	
MN365544.1	2016	Kenya	RSVB	
MN365545.1	2016	Kenya	RSVB	
MN365546.1	2016	Kenya	RSVB	
MN365547.1	2016	Kenya	RSVB	
MN365549.1	2016	Kenya	RSVB	
MN365551.1	2016	Kenya	RSVB	
MN365552.1	2016	Kenya	RSVB	
MN365555.1	2017	Kenya	RSVB	
MN365556.1	2017	Kenya	RSVB	
MN365557.1	2017	Kenya	RSVB	
MN365558.1	2017	Kenya	RSVB	
MN365559.1	2017	Kenya	RSVB	
MN365560.1	2017	Kenya	RSVB	
MN365561.1	2017	Kenya	RSVB	
MN365562.1	2017	Kenya	RSVB	
MN365563.1	2017	Kenya	RSVB	
MN365564.1	2017	Kenya	RSVB	
MN365565.1	2017	Kenya	RSVB	
MN365567.1	2017	Kenya	RSVB	
MN365569.1	2015	Kenya	RSVB	B6
MN365570.1	2016	Kenya	RSVB	
MN365571.1	2016	Kenya	RSVB	
MN365572.1	2016	Kenya	RSVB	
MN365573.1	2016	Kenya	RSVB	
MN365577.1	2016	Kenya	RSVB	
MN365578.1	2016	Kenya	RSVB	
MN365579.1	2016	Kenya	RSVB	
MN365580.1	2016	Kenya	RSVB	
MN365581.1	2016	Kenya	RSVB	
MN365582.1	2016	Kenya	RSVB	
MN365583.1	2016	Kenya	RSVB	
MN365584.1	2017	Kenya	RSVB	
MN365585.1	2017	Kenya	RSVB	
MN365586.1	2017	Kenya	RSVB	
MN365587.1	2017	Kenya	RSVB	
MN365588.1	2017	Kenya	RSVB	
MN365589.1	2017	Kenya	RSVB	
MN365590.1	2017	Kenya	RSVB	
MN365591.1	2017	Kenya	RSVB	
MN365592.1	2017	Kenya	RSVB	
MN365593.1	2017	Kenya	RSVB	

MN365594.1	2017	Kenya	RSVB		
MN365595.1	2017	Kenya	RSVB		
MN365596.1	2017	Kenya	RSVB		
MN365597.1	2017	Kenya	RSVB		
MN365598.1	2017	Kenya	RSVB		
MN365599.1	2017	Kenya	RSVB		
MN365600.1	2017	Kenya	RSVB		
MN531557.1	2013	USA	RSVA		A23
MN630088.1	2016	USA	RSVA		A23
MN630089.1	2016	USA	RSVA		A23
MN630090.1	2016	USA	RSVA		A23
MN630092.1	2016	USA	RSVA		A23
MN630093.1	2016	USA	RSVA		A23
MN630096.1	2016	USA	RSVA		A23
MN630097.1	2016	USA	RSVA		A23
MN630098.1	2016	USA	RSVA		A23
MN630099.1	2016	USA	RSVA		A23
MN630100.1	2016	USA	RSVA		A23
MN630101.1	2016	USA	RSVA		A23
MN630102.1	2016	USA	RSVA		A23
MN630103.1	2016	USA	RSVA		A23
MN630104.1	2016	USA	RSVA		A23
MN630105.1	2016	USA	RSVA		A23
MN630106.1	2016	USA	RSVA		A23
MN630107.1	2016	USA	RSVA		A23
MT040081.1	2016	USA	RSVB		
MT040084.1	2016	USA	RSVB		
MT040085.1	2016	USA	RSVB		
MT040086.1	2016	USA	RSVB		
MT040087.1	2016	USA	RSVB		
MT040088.1	2016	USA	RSVB		
MT040089.1	2016	USA	RSVB		
MT107528.1	2019	Switzerland	RSVB		
MT373703.1	2019	Russia	RSVB		
MT373704.1	2019	Russia	RSVB		
MT373705.1	2019	Russia	RSVB		
MT422269.1	2019	Russia	RSVA		A23
MT422270.1	2019	Russia	RSVA		A23
MT422271.1	2019	Russia	RSVA		A23
MT422272.1	2019	Russia	RSVA		A23
MT422273.1	2019	Russia	RSVA		A23
NC_001781.1		USA	RSVB		B3
U39662.1		UK	RSVA		A4
101_S76	2018	Australia	RSVA	ON1	A23
102_S68	2018	Australia	RSVA	ON1	A23
103K_S115	2018	Australia	RSVA	ON1	A23
103_S29	2018	Australia	RSVA	ON1	A23

116R_S65	2018	Australia	RSVA	ON1	A23
119_17R_S98	2017	Australia	RSVA	ON1	A23
119R_S32	2018	Australia	RSVA		A23
11G_S55	2017	Australia	RSVA		A23
11_S89	2017	Australia	RSVA	ON1	A23
120_S77	2018	Australia	RSVA	ON1	
121_S119	2018	Australia	RSVA	ON1	A23
122_S53	2018	Australia	RSVA	ON1	A23
123_S45	2018	Australia	RSVA	ON1	A23
125_S81	2018	Australia	RSVA	ON1	A23
126_16R_S86	2016	Australia	RSVA	ON1	A23
129_S35	2018	Australia	RSVA	ON1	A23
12_S16	2016	Australia	RSVA	ON1	A23
132_S58	2018	Australia	RSVA	ON1	A23
134_S4	2016	Australia	RSVA	ON1	A23
13G_S18	2018	Australia	RSVA		A23
13_S39	2018	Australia	RSVA	ON1	A23
168_17R_S42	2017	Australia	RSVA	ON1	A23
186_S88	2018	Australia	RSVA	ON1	A23
188_S30	2018	Australia	RSVA	ON1	A23
18_S38	2018	Australia	RSVA	ON1	A23
190_S59	2018	Australia	RSVA	ON1	A23
191_S67	2018	Australia	RSVA	ON1	A23
192_17R_S120	2017	Australia	RSVA	ON1	A23
194_S27	2018	Australia	RSVA	ON1	A23
196_S80	2018	Australia	RSVA	ON1	A23
202_S64	2018	Australia	RSVA	ON1	A23
205_S5	2018	Australia	RSVA	ON1	A23
206_S92	2018	Australia	RSVA	ON1	A23
207_S10	2018	Australia	RSVA	ON1	
208_S116	2018	Australia	RSVA	ON1	A23
210_S70	2018	Australia	RSVA	ON1	A23
211_S31	2016	Australia	RSVA	ON1	A23
212_S26	2018	Australia	RSVA	ON1	A23
213_S125	2018	Australia	RSVA	ON1	A23
214_S71	2017	Australia	RSVA	ON1	A23
215_S100	2018	Australia	RSVA	ON1	A23
544_18R_S126	2018	Australia	RSVA	ON1	A23
55_S17	2016	Australia	RSVA	ON1	A23
57_16R_S90	2016	Australia	RSVA	ON1	
62_S8	2017	Australia	RSVA	ON1	A23
648_16R_S97	2016	Australia	RSVA	ON1	A23
654_16R_S21	2016	Australia	RSVA	ON1	A23
68_S46	2017	Australia	RSVA	ON1	A23
73_S105	2018	Australia	RSVA	ON1	A23
7545B_S6	2018	Australia	RSVA		
7545C_S47	2018	Australia	RSVA	ON1	A23

82_S12	2018	Australia	RSVA	ON1	A23
8399_18_S117	2018	Australia	RSVA	ON1	
87_S94	2018	Australia	RSVA	ON1	A23
88_S37	2018	Australia	RSVA	ON1	A23
8_S85	2018	Australia	RSVA	ON1	A23
92_S57	2018	Australia	RSVA	ON1	A23
93_S34	2018	Australia	RSVA	ON1	A23
94_S52	2018	Australia	RSVA	ON1	A23
96_S54	2018	Australia	RSVA	ON1	A23
97_S124	2018	Australia	RSVA	ON1	A23
98_S113	2018	Australia	RSVA	ON1	A23
99_S93	2018	Australia	RSVA	ON1	A23
KF826847	2007	Australia	RSVA	GA5	A10
KF826848	2007	Australia	RSVA	GA2	A18
MH760588	2016	Australia	RSVA	ON1	A23
MH760589	2016	Australia	RSVA	ON1	A23
MH760590	2016	Australia	RSVA	ON1	A23
MH760591	2016	Australia	RSVA	ON1	A23
MH760592	2010	Australia	RSVA	NA1	A23
MH760593	2010	Australia	RSVA	NA1	A23
MH760594	2010	Australia	RSVA	ON1	A23
MH760595	2010	Australia	RSVA	NA1	A23
MH760596	2010	Australia	RSVA	NA1	A23
MH760597	2010	Australia	RSVA	NA1	A23
MH760598	2010	Australia	RSVA	NA1	A23
MH760599	2010	Australia	RSVA	GA5	A11
MH760600	2011	Australia	RSVA	NA1	A23
MH760601	2012	Australia	RSVA	NA1	A23
MH760602	2012	Australia	RSVA	ON1	A23
MH760603	2012	Australia	RSVA	NA1	A23
MH760604	2012	Australia	RSVA	NA1	A23
MH760605	2012	Australia	RSVA	NA1	A23
MH760606	2013	Australia	RSVA	ON1	A23
MH760607	2013	Australia	RSVA	ON1	A23
MH760608	2016	Australia	RSVA	ON1	A23
MH760609	2016	Australia	RSVA	ON1	A23
MH760610	2016	Australia	RSVA	ON1	A23
MH760611	2016	Australia	RSVA	ON1	A23
MH760612	2016	Australia	RSVA	ON1	A23
MH760613	2016	Australia	RSVA	ON1	A23
MH760614	2016	Australia	RSVA	ON1	A23
MH760615	2016	Australia	RSVA	ON1	A23
MH760616	2016	Australia	RSVA	ON1	A23
MH760617	2016	Australia	RSVA	ON1	A23
MH760618	2016	Australia	RSVA	ON1	A23
MH760619	2016	Australia	RSVA	ON1	A23
MH760620	2016	Australia	RSVA	ON1	A23

MH760621	2016	Australia	RSVA	ON1	A23
MH760622	2016	Australia	RSVA	ON1	A23
MH760623	2016	Australia	RSVA	ON1	A23
MH760624	2016	Australia	RSVA	ON1	A23
MH760625	2016	Australia	RSVA	ON1	A23
MH760626	2016	Australia	RSVA	ON1	A23
MH760627	2016	Australia	RSVA	ON1	A23
MH760628	2016	Australia	RSVA	ON1	A23
MH760629	2016	Australia	RSVA	ON1	A23
MH760630	2016	Australia	RSVA	ON1	A23
MH760631	2016	Australia	RSVA	ON1	A23
MH760632	2016	Australia	RSVA	ON1	A23
MH760633	2016	Australia	RSVA	ON1	A23
MH760634	2016	Australia	RSVA	ON1	A23
MH760635	2016	Australia	RSVA	ON1	A23
MH760636	2016	Australia	RSVA	ON1	A23
MH760637	2016	Australia	RSVA	ON1	A23
MH760638	2016	Australia	RSVA	ON1	A23
MH760639	2016	Australia	RSVA	ON1	A23
MH760640	2014	Australia	RSVA	ON1	A23
MH760641	2014	Australia	RSVA	NA1	A23
MH760642	2014	Australia	RSVA	ON1	A23
MH760643	2014	Australia	RSVA	ON1	A23
MH760644	2014	Australia	RSVA	ON1	A23
MH760645	2014	Australia	RSVA	ON1	A23
MH760646	2014	Australia	RSVA	ON1	A23
MH760647	2015	Australia	RSVA	ON1	A23
MH760648	2015	Australia	RSVA	ON1	A23
MH760649	2015	Australia	RSVA	ON1	A23
MH760650	2015	Australia	RSVA	ON1	A23
MH760651	2015	Australia	RSVA	ON1	A23
MW020596	2018	Australia	RSVA	ON1	A23
MW020597	2018	Australia	RSVA	ON1	A23
MW020598	2017	Australia	RSVA	ON1	A23
MW020599	2017	Australia	RSVA	ON1	A23
MW160746	2016	Australia	RSVA	ON1	A23
MW160747	2016	Australia	RSVA	ON1	A23
MW160749	2016	Australia	RSVA	ON1	A23
MW160753	2016	Australia	RSVA	ON1	A23
MW160754	2016	Australia	RSVA	ON1	A23
MW160756	2016	Australia	RSVA	ON1	A23
MW160758	2016	Australia	RSVA	ON1	A23
MW160759	2016	Australia	RSVA	ON1	A23
MW160761	2017	Australia	RSVA	ON1	A23
MW160762	2017	Australia	RSVA	ON1	A23
MW160764	2017	Australia	RSVA	ON1	A23
MW160766	2016	Australia	RSVA	ON1	A23

MW160767	2017	Australia	RSVA	ON1	A23
MW160768	2017	Australia	RSVA	ON1	A23
MW160769	2017	Australia	RSVA	ON1	A23
MW160771	2017	Australia	RSVA	ON1	A23
MW160772	2017	Australia	RSVA	ON1	A23
MW160773	2017	Australia	RSVA	ON1	A23
MW160774	2017	Australia	RSVA	ON1	A23
MW160776	2017	Australia	RSVA	ON1	A23
MW160778	2017	Australia	RSVA	ON1	A23
MW160779	2017	Australia	RSVA	ON1	A23
MW160782	2017	Australia	RSVA	ON1	A23
MW160783	2017	Australia	RSVA	ON1	A23
MW160785	2017	Australia	RSVA	ON1	A23
MW160787	2017	Australia	RSVA	ON1	A23
MW160795	2016	Australia	RSVA	ON1	A23
MW160797	2016	Australia	RSVA	ON1	A23
MW160799	2016	Australia	RSVA	ON1	A23
MW160801	2016	Australia	RSVA	ON1	A23
MW160803	2016	Australia	RSVA	ON1	A23
MW160807	2017	Australia	RSVA	ON1	A23
MW160809	2017	Australia	RSVA	ON1	A23
MW160819	2017	Australia	RSVA	ON1	A23
MW160820	2017	Australia	RSVA	ON1	A23
MW160822	2017	Australia	RSVA	ON1	A23
MW160824	2017	Australia	RSVA	ON1	A23
MW160825	2017	Australia	RSVA	ON1	A23
MW160827	2017	Australia	RSVA	ON1	A23
MW160828	2017	Australia	RSVA	ON1	A23
MW160830	2017	Australia	RSVA	ON1	A23
VR26E_S110	1956		RSVA		
VR26G_S114	1956		RSVA		
VR26K_S102	1956		RSVA		
128_S24	2018	Australia	RSVB	BA10	B6
139_S11	2018	Australia	RSVB	BA10	B6
141_S43	2018	Australia	RSVB	BA10	B6
142_S107	2018	Australia	RSVB	BA10	B6
146_S87	2018	Australia	RSVB	BA10	B6
150_S78	2018	Australia	RSVB	BA10	B6
154_S106	2018	Australia	RSVB	BA10	B6
156_S22	2017	Australia	RSVB	BA10	B6
157_S118	2018	Australia	RSVB	BA10	B6
158_S66	2018	Australia	RSVB	BA10	B6
163_S9	2017	Australia	RSVB	BA10	B6
165_S19	2017	Australia	RSVB	BA10	B6
166_S123	2018	Australia	RSVB	BA10	B6
169_S13	2018	Australia	RSVB	BA10	B6
170_S122	2018	Australia	RSVB	BA10	B6

171_S2	2018	Australia	RSVB	BA10	B6
174_S51	2018	Australia	RSVB	BA10	B6
175_S40	2018	Australia	RSVB	BA10	B6
176_S41	2017	Australia	RSVB	BA10	B6
177_S62	2018	Australia	RSVB	BA10	B6
178_S75	2016	Australia	RSVB	BA10	B6
183_S3	2018	Australia	RSVB	BA10	B6
187_S44	2018	Australia	RSVB	BA10	B6
192_S83	2018	Australia	RSVB	BA10	B6
193_S72	2018	Australia	RSVB	BA10	B6
195_S61	2018	Australia	RSVB	BA10	B6
19C_S20	2018	Australia	RSVB	BA10	B6
200_S56	2018	Australia	RSVB	BA10	B6
201G_S15	2018	Australia	RSVB	BA10	B6
201_S23	2018	Australia	RSVB	BA10	B6
203_S33	2018	Australia	RSVB	BA10	B6
204_S96	2018	Australia	RSVB	BA10	B6
33_S25	2018	Australia	RSVB	BA10	B6
36_S74	2018	Australia	RSVB	BA10	B6
39_S121	2018	Australia	RSVB	BA10	B6
4104_17R_S108	2017	Australia	RSVB	BA10	B6
42_S14	2017	Australia	RSVB	BA10	B6
43_S109	2018	Australia	RSVB	BA10	B6
44_S60	2016	Australia	RSVB	BA10	B6
48_S49	2017	Australia	RSVB	BA10	B6
516_16R_S99	2016	Australia	RSVB	BA10	B6
59_S112	2017	Australia	RSVB	BA10	B6
616_18R_S69	2018	Australia	RSVB	BA10	B6
693_17R_S104	2017	Australia	RSVB	BA10	B6
69_S103	2016	Australia	RSVB	BA10	B6
70_S63	2018	Australia	RSVB	BA10	B6
714_17R_S82	2017	Australia	RSVB	BA10	B6
7172_17R_S73	2017	Australia	RSVB	BA10	B6
71_S50	2018	Australia	RSVB	BA10	B6
732_17R_S95	2017	Australia	RSVB	BA10	B6
75_S1	2016	Australia	RSVB	BA10	B6
78_S36	2018	Australia	RSVB	BA10	B6
79B_S48	2017	Australia	RSVB	BA10	B6
80_S79	2018	Australia	RSVB	BA10	B6
8180_17R_S111	2017	Australia	RSVB	BA10	B6
83A_S101	2018	Australia	RSVB	BA10	B6
MH760652	2016	Australia	RSVB	BA9	B6
MH760653	2015	Australia	RSVB	BA10	B6
MH760654	2015	Australia	RSVB	BA10	B6
MH760655	2010	Australia	RSVB	BA9	B6
MH760656	2010	Australia	RSVB	BA9	B6
MH760657	2010	Australia	RSVB	BA9	B6

MH760658	2010	Australia	RSVB	BA9	B6
MH760659	2011	Australia	RSVB	BA9	B6
MH760660	2012	Australia	RSVB	BA9	B6
MH760661	2012	Australia	RSVB	BA9	B6
MH760662	2012	Australia	RSVB	BA9	B6
MH760663	2012	Australia	RSVB	BA9	B6
MH760664	2012	Australia	RSVB	BA9	B6
MH760665	2013	Australia	RSVB	BA9	B6
MH760666	2013	Australia	RSVB	BA9	B6
MH760667	2013	Australia	RSVB	BA9	B6
MH760668	2016	Australia	RSVB	BA10	B6
MH760669	2016	Australia	RSVB	BA10	B6
MH760670	2016	Australia	RSVB	BA10	B6
MH760671	2016	Australia	RSVB	BA10	B6
MH760672	2016	Australia	RSVB	BA10	B6
MH760673	2016	Australia	RSVB	BA10	B6
MH760674	2016	Australia	RSVB	BA10	B6
MH760675	2016	Australia	RSVB	BA10	B6
MH760676	2016	Australia	RSVB	BA10	B6
MH760677	2016	Australia	RSVB	BA10	B6
MH760678	2016	Australia	RSVB	BA10	B6
MH760679	2016	Australia	RSVB	BA10	B6
MH760680	2016	Australia	RSVB	BA10	B6
MH760681	2016	Australia	RSVB	BA10	B6
MH760682	2016	Australia	RSVB	BA10	B6
MH760683	2016	Australia	RSVB	BA10	B6
MH760684	2016	Australia	RSVB	BA10	B6
MH760685	2016	Australia	RSVB	BA10	B6
MH760686	2016	Australia	RSVB	BA10	B6
MH760687	2016	Australia	RSVB	BA10	B6
MH760688	2016	Australia	RSVB	BA10	B6
MH760689	2016	Australia	RSVB	BA10	B6
MH760690	2016	Australia	RSVB	BA10	B6
MH760691	2016	Australia	RSVB	BA10	B6
MH760692	2016	Australia	RSVB	BA10	B6
MH760693	2016	Australia	RSVB	BA10	B6
MH760694	2016	Australia	RSVB	BA10	B6
MH760695	2016	Australia	RSVB	BA10	B6
MH760696	2016	Australia	RSVB	BA10	B6
MH760697	2016	Australia	RSVB	BA9	B6
MH760698	2016	Australia	RSVB	BA10	B6
MH760699	2016	Australia	RSVB	BA10	B6
MH760700	2016	Australia	RSVB	BA10	B6
MH760701	2016	Australia	RSVB	BA10	B6
MH760702	2016	Australia	RSVB	BA10	B6
MH760703	2016	Australia	RSVB	BA10	B6
MH760704	2016	Australia	RSVB	BA10	B6

MH760705	2016	Australia	RSVB	BA10	B6
MH760706	2016	Australia	RSVB	BA10	B6
MH760707	2016	Australia	RSVB	BA10	B6
MH760708	2016	Australia	RSVB	BA10	B6
MH760709	2016	Australia	RSVB	BA10	B6
MH760710	2016	Australia	RSVB	BA10	B6
MH760711	2016	Australia	RSVB	BA10	B6
MH760712	2016	Australia	RSVB	BA10	B6
MH760713	2016	Australia	RSVB	BA10	B6
MH760714	2016	Australia	RSVB	BA10	B6
MH760715	2016	Australia	RSVB	BA10	B6
MH760716	2016	Australia	RSVB	BA10	B6
MH760717	2016	Australia	RSVB	BA10	B6
MH760718	2016	Australia	RSVB	BA10	B6
MH760719	2016	Australia	RSVB	BA9	B6
MH760720	2016	Australia	RSVB	BA10	B6
MH760721	2016	Australia	RSVB	BA10	B6
MH760722	2016	Australia	RSVB	BA10	B6
MH760723	2016	Australia	RSVB	BA10	B6
MH760724	2013	Australia	RSVB	BA9	B6
MH760725	2014	Australia	RSVB	BA10	B6
MH760726	2015	Australia	RSVB	BA10	B6
MH760727	2015	Australia	RSVB	BA9	B6
MH760728	2015	Australia	RSVB	BA10	B6
MH760729	2015	Australia	RSVB	BA10	B6
MH760730	2015	Australia	RSVB	BA9	B6
MH760731	2015	Australia	RSVB	BA10	B6
MW020595	2018	Australia	RSVB	BA10	B6
MW160744	2016	Australia	RSVB	BA10	B6
MW160745	2016	Australia	RSVB	BA10	B6
MW160748	2016	Australia	RSVB	BA10	B6
MW160750	2016	Australia	RSVB	BA10	B6
MW160751	2016	Australia	RSVB	BA10	B6
MW160752	2016	Australia	RSVB	BA10	B6
MW160755	2016	Australia	RSVB	BA10	B6
MW160757	2016	Australia	RSVB	BA10	B6
MW160760	2016	Australia	RSVB	BA10	B6
MW160763	2017	Australia	RSVB	BA10	B6
MW160765	2017	Australia	RSVB	BA10	B6
MW160770	2017	Australia	RSVB	BA10	B6
MW160775	2017	Australia	RSVB	BA10	B6
MW160777	2017	Australia	RSVB	BA10	B6
MW160780	2017	Australia	RSVB	BA10	B6
MW160781	2017	Australia	RSVB	BA10	B6
MW160784	2017	Australia	RSVB	BA10	B6
MW160786	2017	Australia	RSVB	BA10	B6
MW160788	2016	Australia	RSVB	BA10	B6

MW160789	2016	Australia	RSVB	BA10	B6
MW160790	2016	Australia	RSVB	BA10	B6
MW160791	2016	Australia	RSVB	BA10	B6
MW160792	2016	Australia	RSVB	BA10	B6
MW160793	2016	Australia	RSVB	BA10	B6
MW160794	2016	Australia	RSVB	BA10	B6
MW160796	2016	Australia	RSVB	BA10	B6
MW160798	2016	Australia	RSVB	BA10	B6
MW160800	2016	Australia	RSVB	BA10	B6
MW160802	2016	Australia	RSVB	BA10	B6
MW160804	2016	Australia	RSVB	BA10	B6
MW160805	2016	Australia	RSVB	BA10	B6
MW160806	2016	Australia	RSVB	BA10	B6
MW160808	2017	Australia	RSVB	BA10	B6
MW160810	2017	Australia	RSVB	BA10	B6
MW160811	2017	Australia	RSVB	BA10	B6
MW160812	2017	Australia	RSVB	BA10	B6
MW160813	2017	Australia	RSVB	BA10	B6
MW160814	2017	Australia	RSVB	BA10	B6
MW160815	2017	Australia	RSVB	BA10	B6
MW160816	2017	Australia	RSVB	BA10	B6
MW160817	2017	Australia	RSVB	BA10	B6
MW160818	2017	Australia	RSVB	BA10	B6
MW160821	2017	Australia	RSVB	BA10	B6
MW160823	2017	Australia	RSVB	BA10	B6
MW160826	2017	Australia	RSVB	BA10	B6
MW160829	2017	Australia	RSVB	BA10	B6
MW160831	2017	Australia	RSVB	BA10	B6
MW160832	2017	Australia	RSVB	BA10	B6
MW160833	2017	Australia	RSVB	BA10	B6
MW160834	2017	Australia	RSVB	BA10	B6
MW160835	2017	Australia	RSVB	BA10	B6
MW160836	2017	Australia	RSVB	BA10	B6
MW160837	2017	Australia	RSVB	BA10	B6
MW160838	2017	Australia	RSVB	BA10	B6
MW160839	2017	Australia	RSVB	BA10	B6
VR955E_S84	1977		RSVB		
VR955G_S7	1977		RSVB		

Supplementary Figures

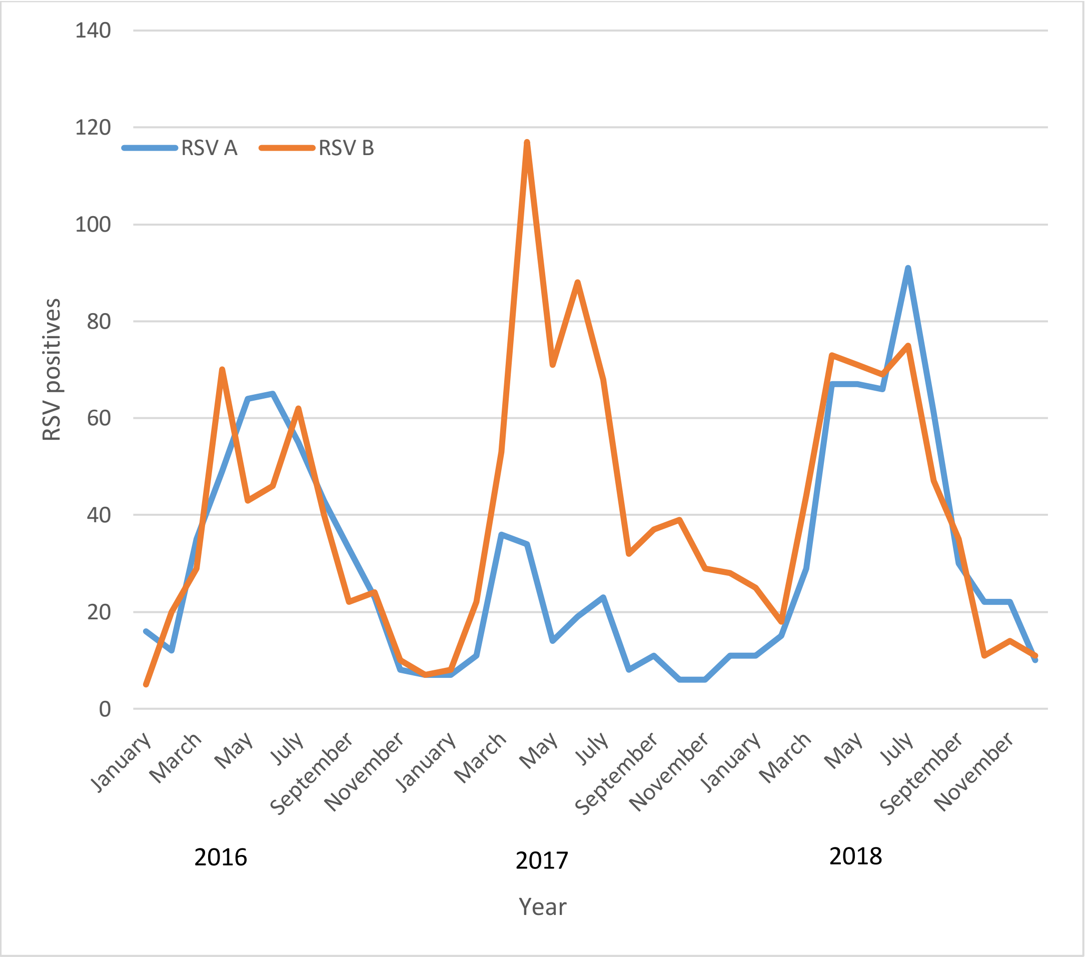

Supplementary Figure 1. Distribution of RSV subgroups from Westmead over three epidemic seasons from January 2016 to December 2018

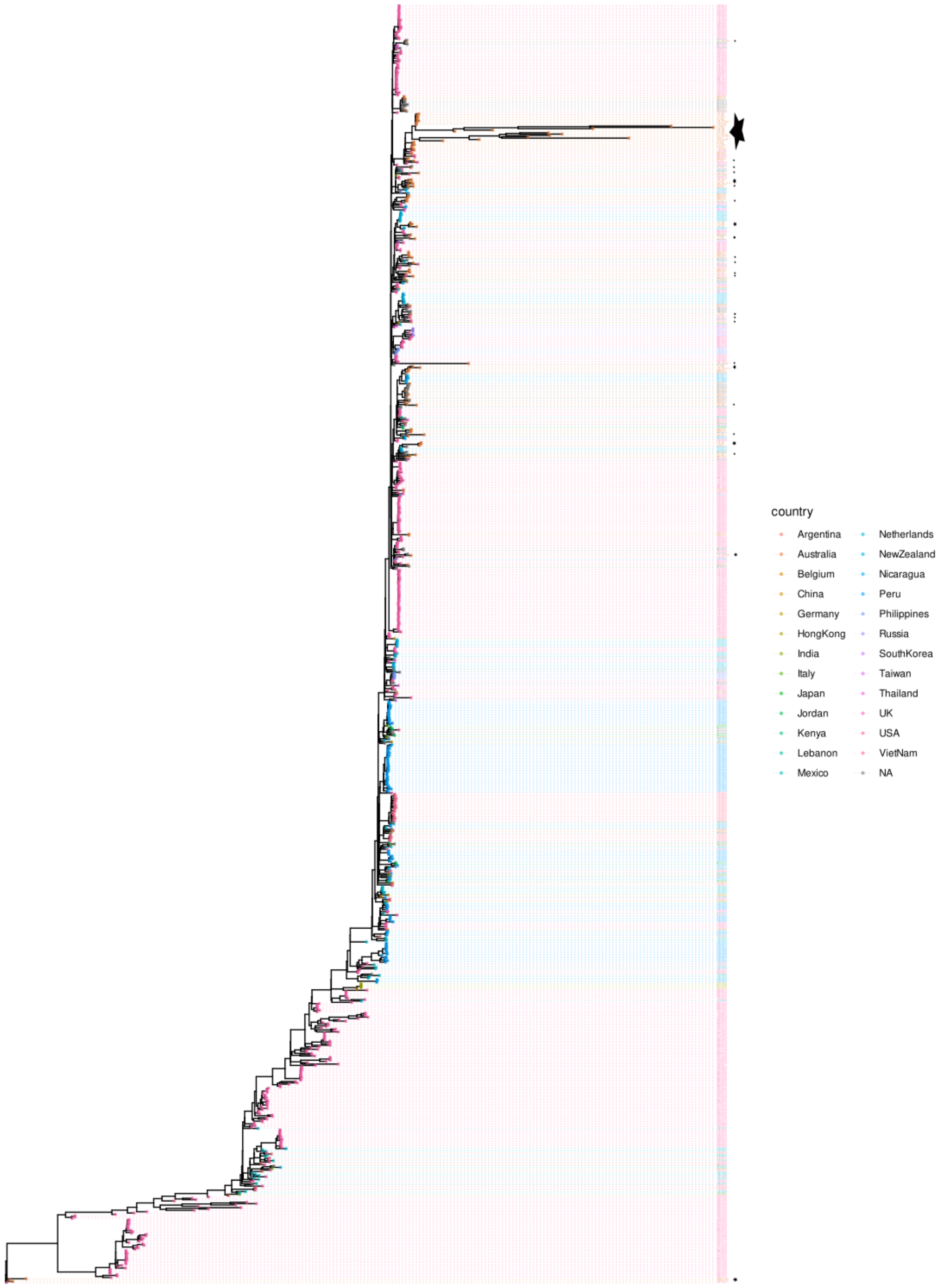

Supplementary Figure 2. Global phylogeny of respiratory syncytial virus subtype A. A mid-point rooted whole-genome based maximum likelihood phylogenomic tree of RSV-A strains sequenced in this study (clades labelled with black stars) and the corresponding published global RSV-A genomes. The strains are coloured according to the location of collection. The branch lengths are scaled according to the number of substitutions per site.

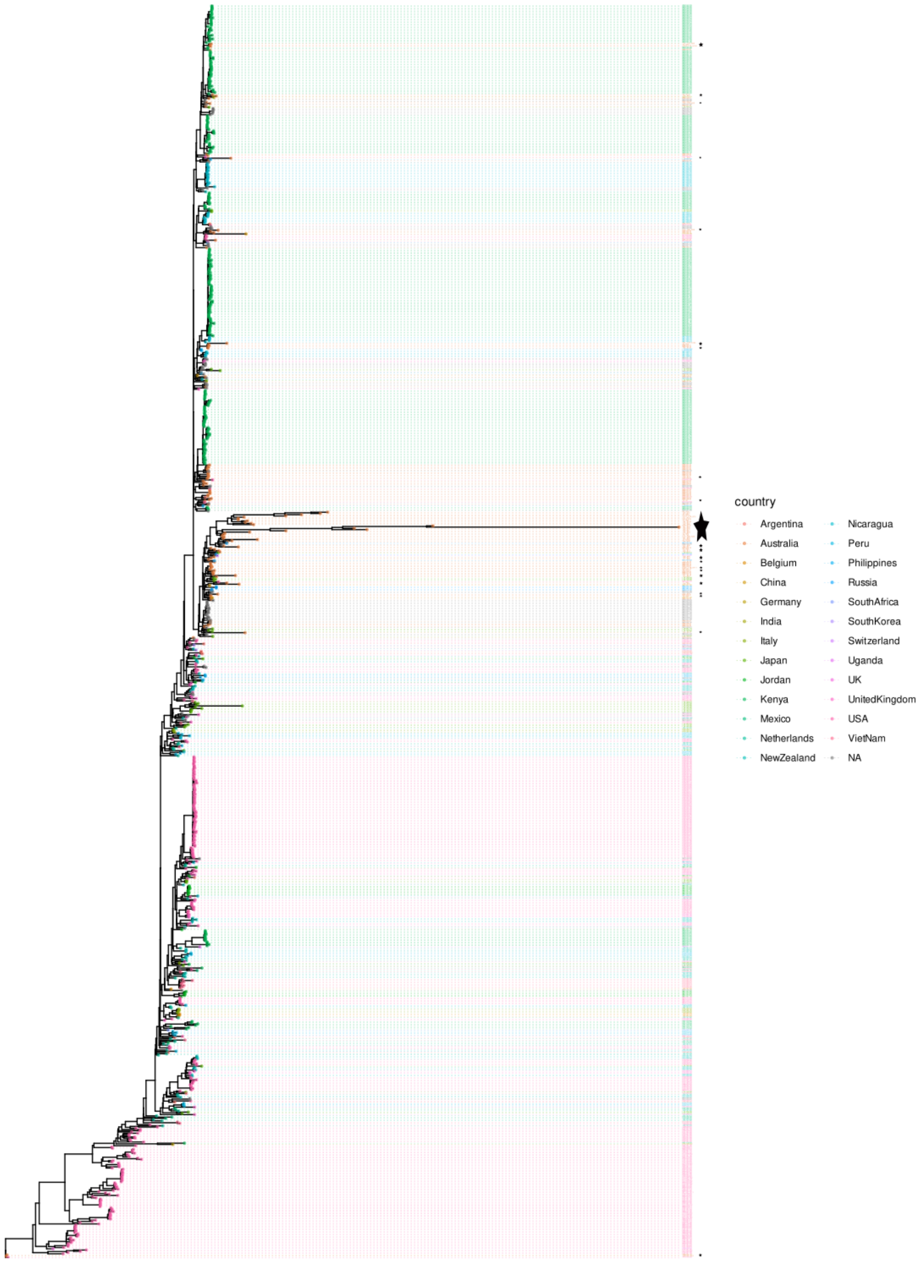

Supplementary Figure 3. Global phylogeny of respiratory syncytial virus subtype B. A mid-point rooted whole-genome based maximum likelihood phylogenomic tree of RSV-B strains sequenced in this study (clades labelled with black stars) and the corresponding published global RSV-B genomes. The strains are coloured according to the location of collection. The branch lengths are scaled according to the number of substitutions per site.

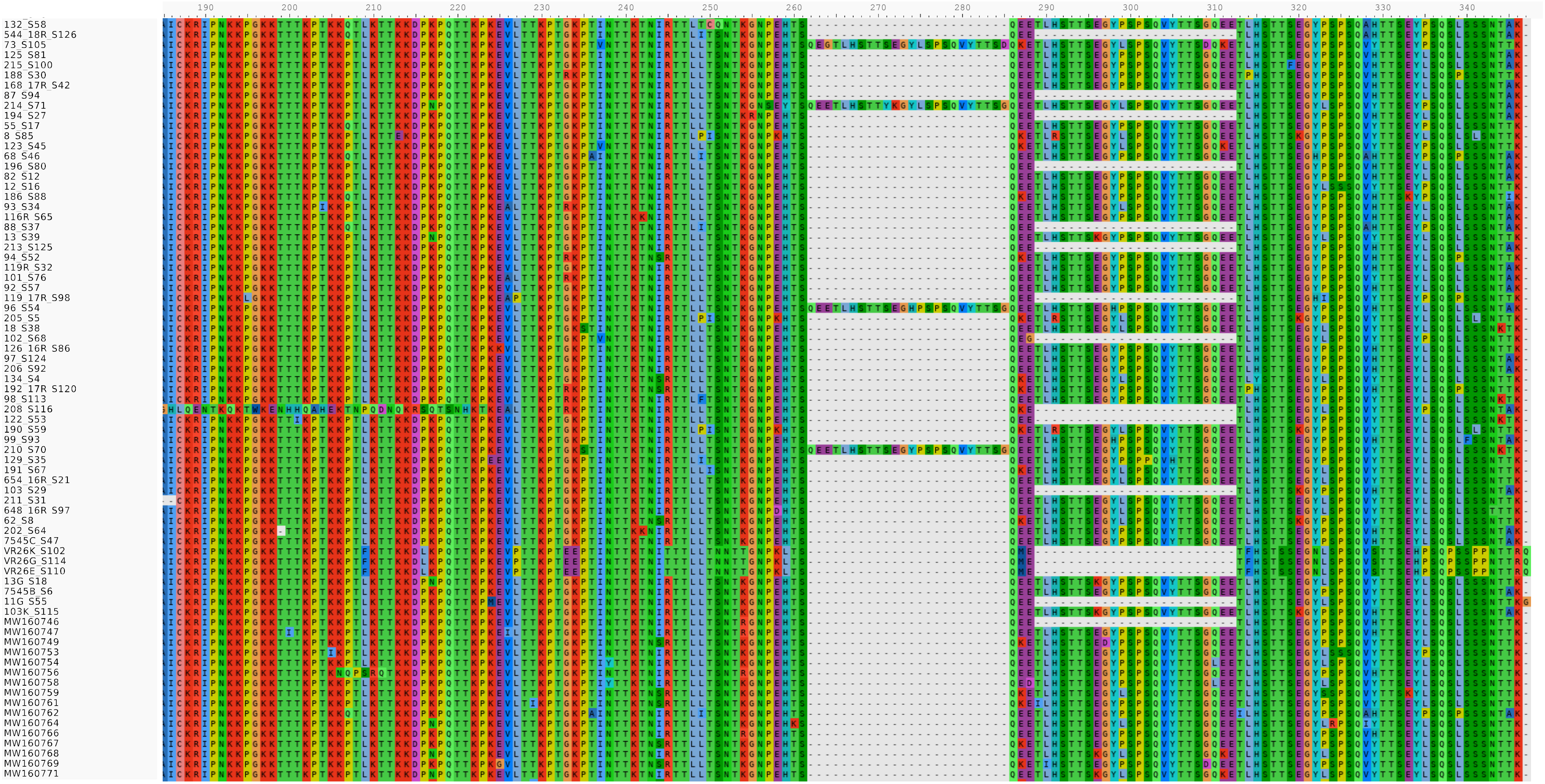

Supplementary Figure 4: Alignment of unique deduced amino acid sequences of the G protein of RSVA isolates. The detection of RSVA ON1 strains with a novel 72-nucleotide triplication (23-amino acid triplication) in the sequence that corresponds to the C-terminal region of the G gene.

A

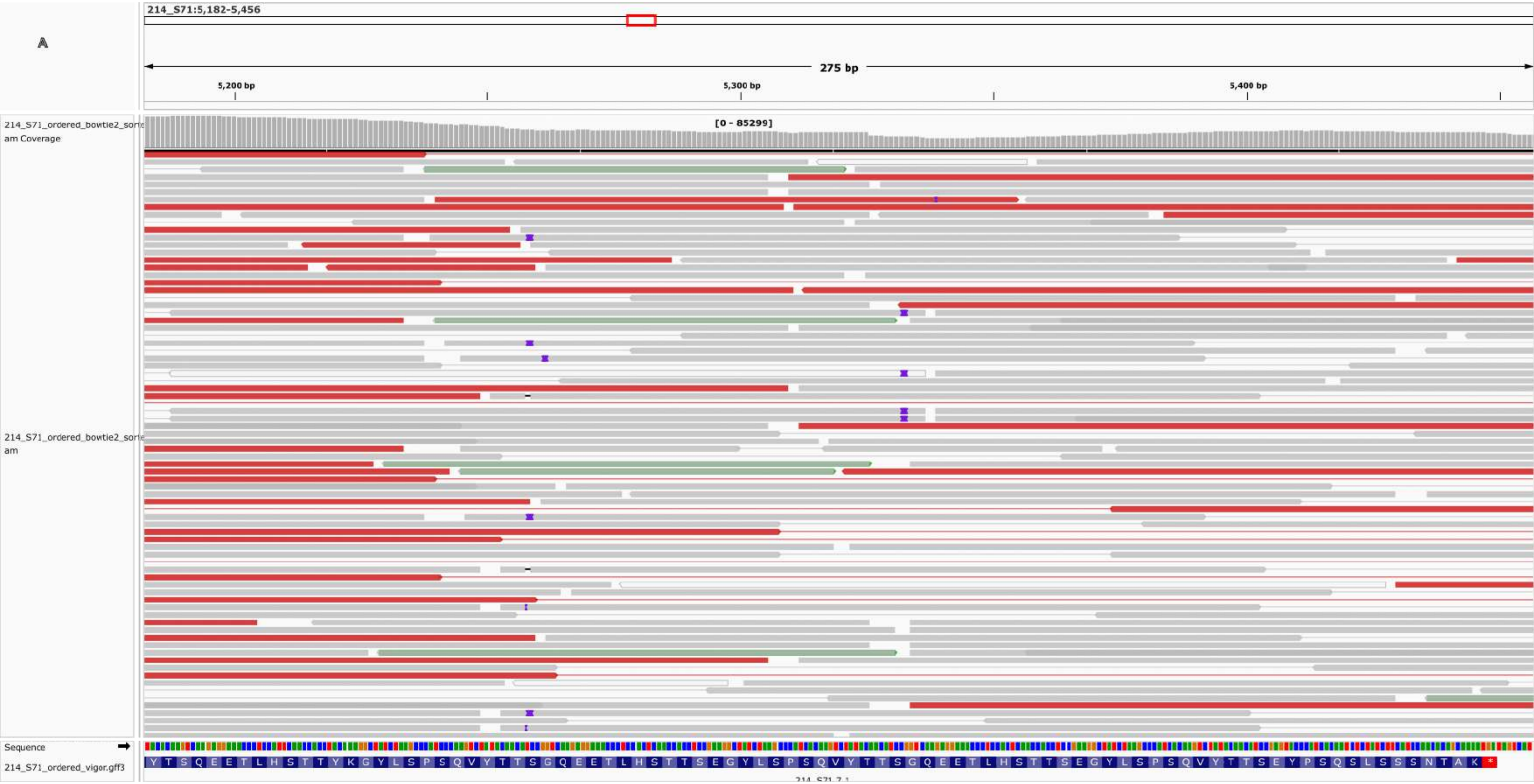
214_S71:5,182-5,456
275 bp
5,200 bp
5,300 bp
5,400 bp
[0 - 85299]
214_S71_ordered_bowtie2_sorte am Coverage
214_S71_ordered_bowtie2_sorte am
Sequence
214_S71_ordered_vigor.gff3

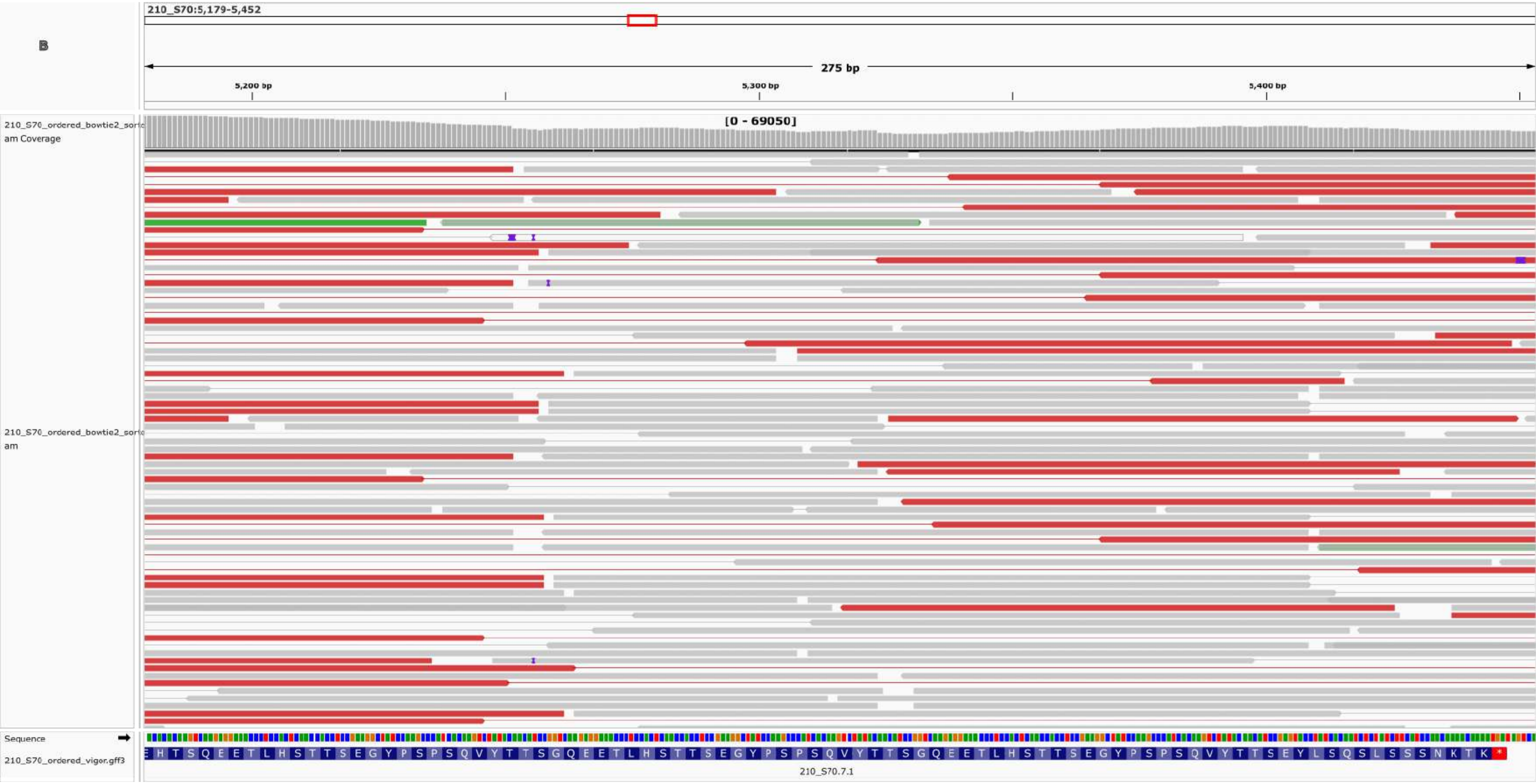

B
210_S70:5,179-5,452
275 bp
5,200 bp
5,300 bp
5,400 bp
210_S70_ordered_bowtie2_sorted.bam Coverage
[0 - 69050]
210_S70_ordered_bowtie2_sorted.bam
Sequence
210_S70_ordered_vigor.gff3
210_S70.7.1

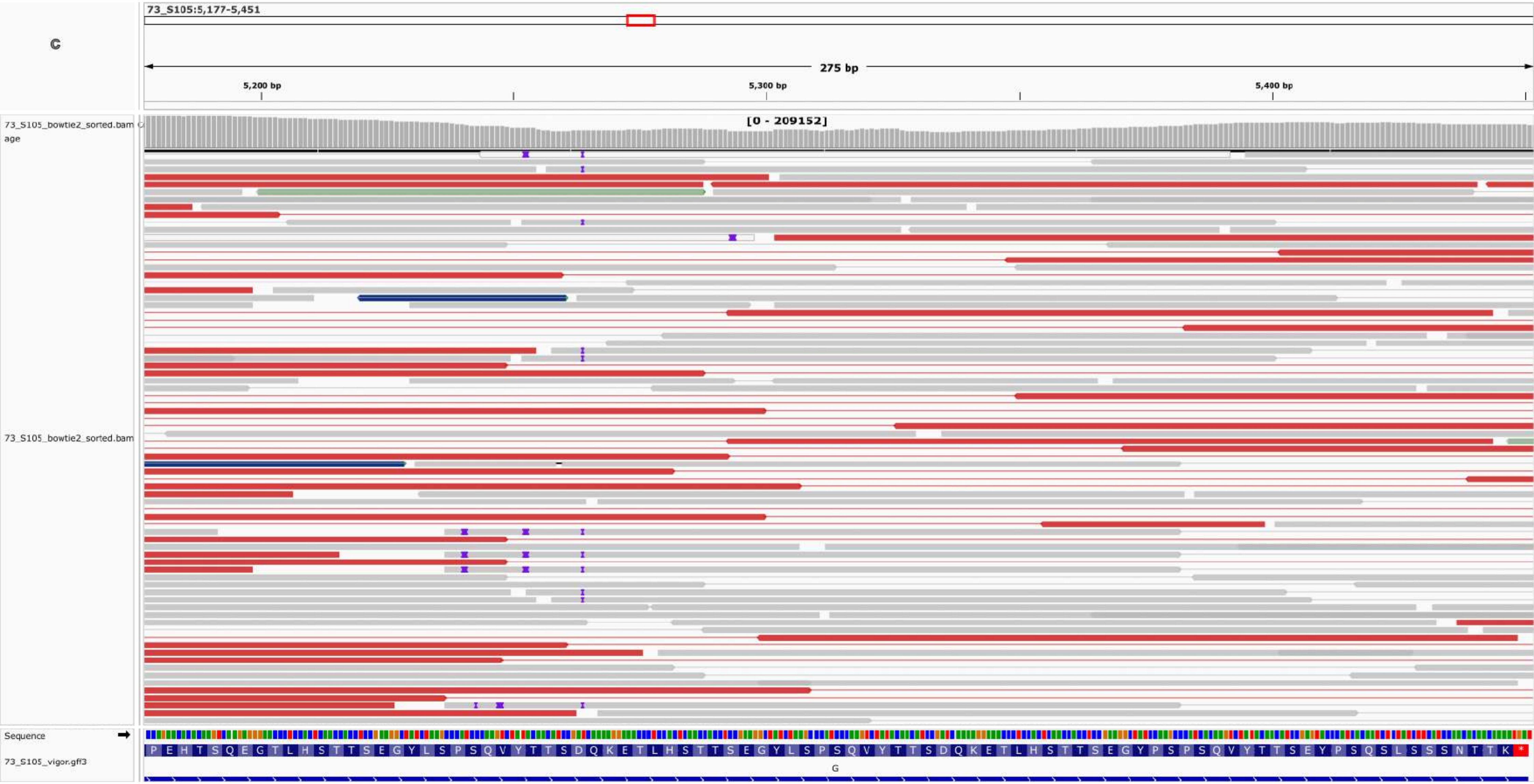
C
73_S105:5,177-5,451
275 bp
5,200 bp
5,300 bp
5,400 bp
73_S105_bowtie2_sorted.bam C
age
[0 - 209152]
73_S105_bowtie2_sorted.bam
Sequence
73_S105_vigor.gff3
P E H T S Q E G T L H S T T S E G Y L S P S Q V Y T T S D Q K E T L H S T T S E G Y L S P S Q V Y T T S D Q K E T L H S T T S E G Y P S P S Q V Y T T S E Y P S Q S L S S S N T T K *
G

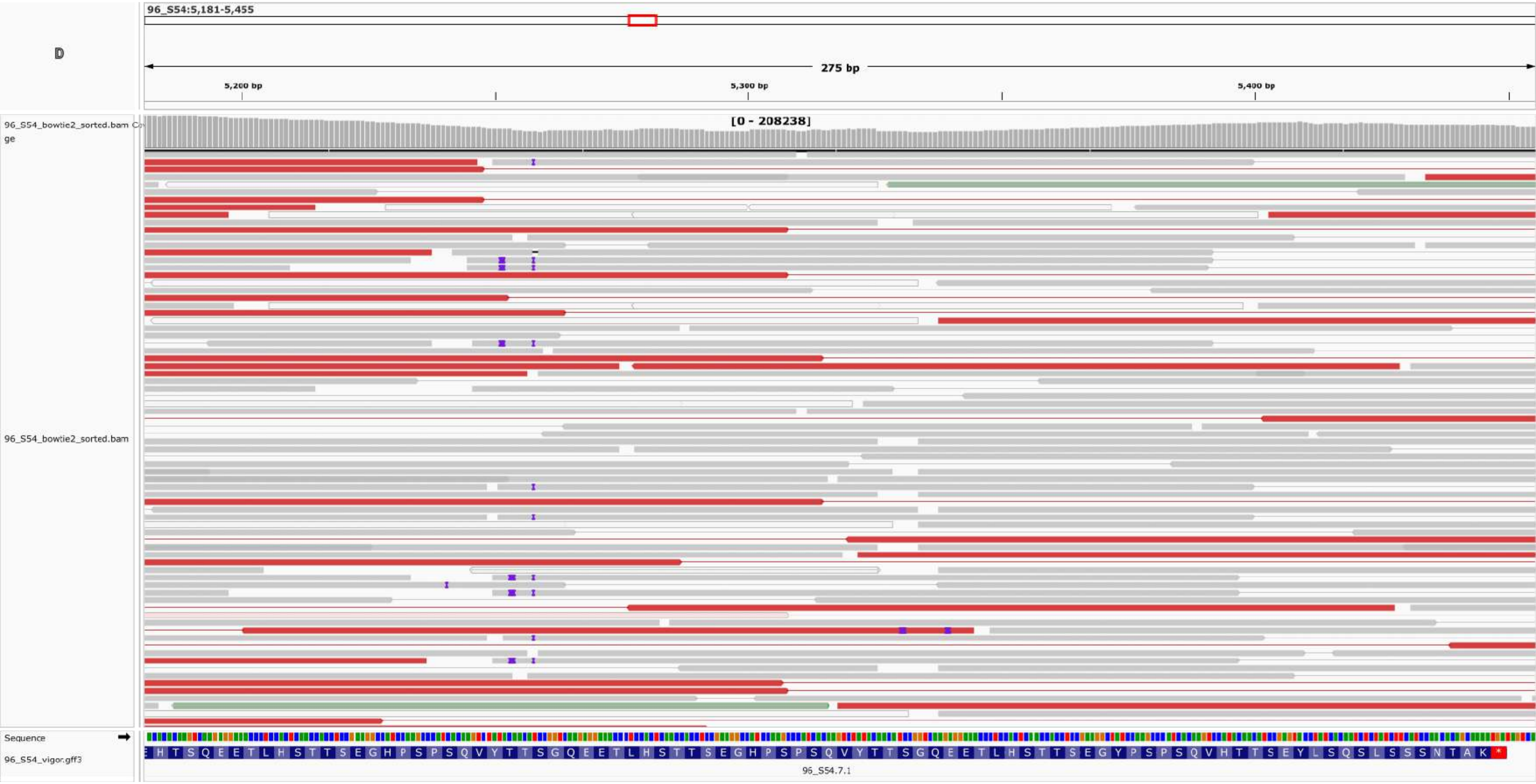
D
96_S54:5,181-5,455
275 bp
5,200 bp
5,300 bp
5,400 bp
[0 - 208238]
96_S54_bowtie2_sorted.bam
Sequence
96_S54_vigor.gff3
96_S54.7.1

Supplementary Figure 5: IGV read mapping view (Collapsed, Read Pair) of representative C-terminal triplication sequences. Sequences 214_S71 (A), 210_S70 (B), 73_S105 (C) and 96_S54 (D) show the triplication region (at the bottom) and read coverage range as numbers in the brackets. Coverage numbers (at Y scale) are showing read counts as a bar chart for each position in the respective region. They are in the range starting from 0 to 85299, 69050, 209152 and 208238 respectively. The reads are colored based on their insert size and orientation.

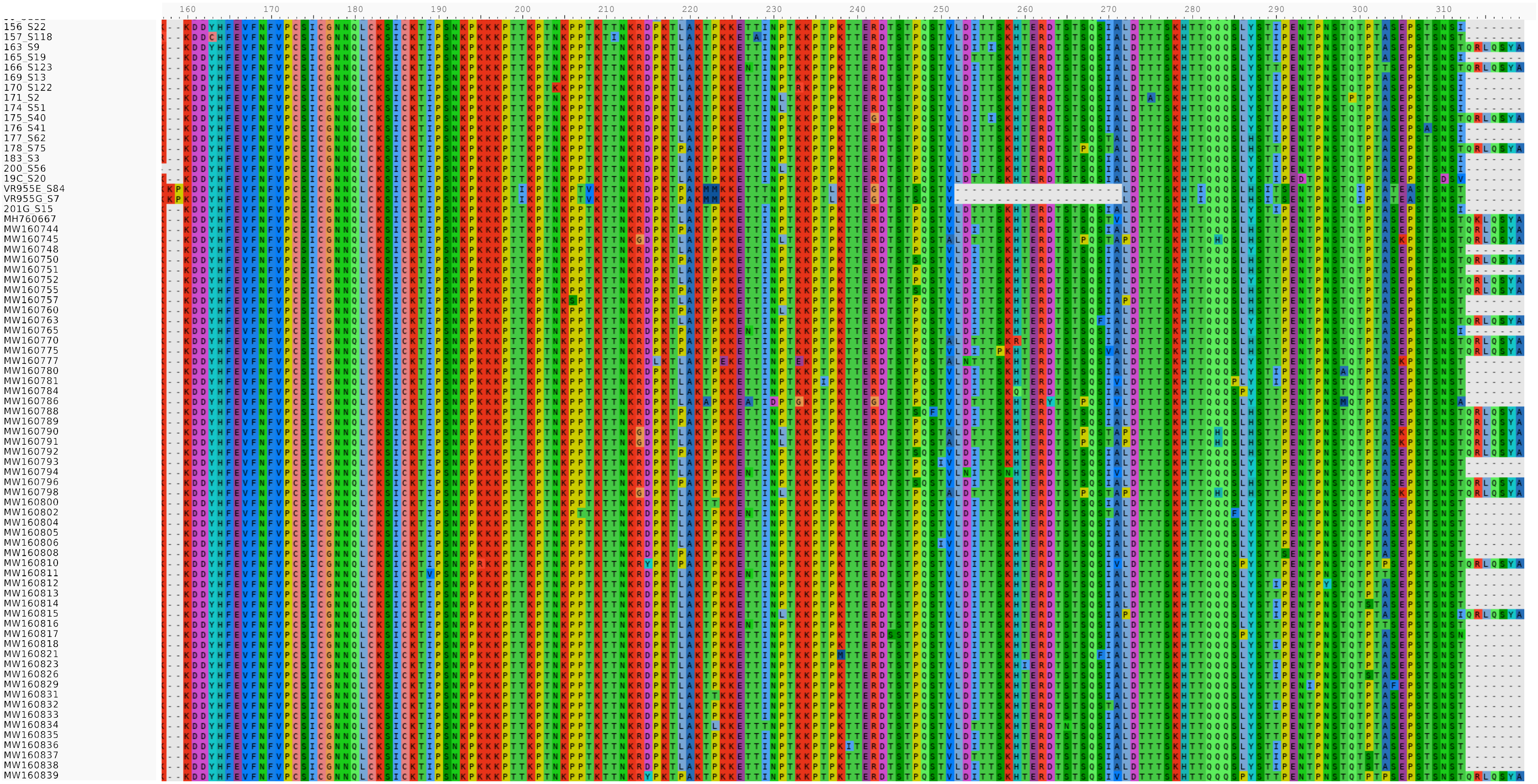

Supplementary Figure 6: Alignment of unique deduced amino acid sequences of the G protein of RSVB isolates. All genomes were characterised by a 60-nucleotide duplication in the second variable region of the G gene.
